# Kendrick Mass Defect Filtering Enables High-Throughput Untargeted Annotation of Minor Phytocannabinoids: Toward Streamlined Phytocannabinomics

**DOI:** 10.1021/acsmeasuresciau.5c00106

**Published:** 2025-10-23

**Authors:** Andrea Cerrato, Giuseppe Cannazza, Cinzia Citti, Aldo Laganà, Roberta Paris, Anna Laura Capriotti

**Affiliations:** † Department of Chemistry, Sapienza University of Rome, Piazzale Aldo Moro 5, 00185 Rome, Italy; ‡ Interuniversity Consortium INBB − Biostructures and Biosystems National Institute, Via dei Carpegna 19, 00165 Rome, Italy; § Department of Life Sciences, 9306 University of Modena and Reggio Emilia, Via Giuseppe Campi 287, 41125 Modena, Italy; ∥ CNR NANOTEC, Campus Ecotekne, University of Salento, Via Monteroni, 73100 Lecce, Italy; ⊥ CREA, Research Centre for Cereal and Industrial Crops, Via di Corticella 133, 40128 Bologna, Italy

**Keywords:** C. sativa, high-resolution mass spectrometry, cannabinoids, compound discoverer, chemovar, geographical origin

## Abstract

Phytocannabinoids are a diverse class of bioactive compounds produced by *Cannabis sativa*, including both major and a growing number of minor constituents with pharmacological relevance. However, their comprehensive annotation in untargeted high-resolution mass spectrometry (HRMS) data sets remains a significant analytical challenge due to their structural similarity, low abundance, and the complexity of plant matrices. In this study, we present a comparative evaluation of Kendrick Mass Defect (KMD)-based filtering workflows for the efficient untargeted annotation of minor phytocannabinoids. Three data processing strategies were implemented using Compound Discoverer: (i) KMD filtering before the “Compound Detection” tool, (ii) KMD filtering after the “Compound Detection” tool, and (iii) a pseudo-KMD approach based on the generation of expected compounds. These workflows were tested and compared using a data set comprising 50 *Cannabis* inflorescence samples analyzed in an untargeted fashion, taking into account the phytocannabinoid coverage, false positive rates, computation burden, and versatility. A total of 61 phytocannabinoids were annotated, including a full series of alkyl homologues (C1–C7), cis/trans isomers, *O*-methylated derivatives, and sesquicannabinoids. Statistical analyses revealed meaningful chemical differentiation based on seed origin, chemovar classification, and reproductive strategy (dioecious vs monoecious), highlighting the biological significance of minor cannabinoids. Overall, the results demonstrate that KMD filtering significantly enhances the throughput and accuracy of untargeted HRMS workflows for structurally related classes of compounds.

## Graphical abstract

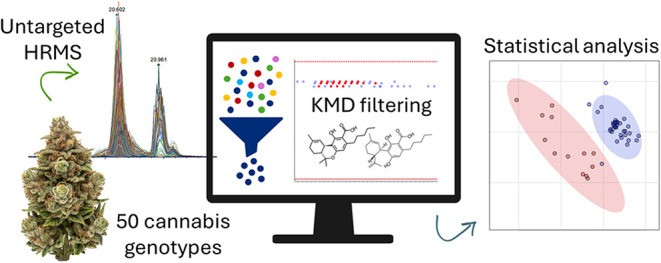

## 1 Introduction

Phytocannabinoids are a broad class of terpenophenolic compounds that are biosynthesized in the glandular trichomes of *C. sativa*, a multipurpose species that has been harvested throughout recorded history, despite remaining a source of controversy even nowadays.^1,2^ The main biosynthetic pathway of phytocannabinoids starts from hexanoyl-CoA, which undergoes a polyketide condensation with three molecules of malonyl-CoA catalyzed by olivetol synthase. This is followed by a subsequent cyclization reaction catalyzed by the olivetolic acid cyclase to obtain olivetolic acid, the phenolic portion of cannabinoids.^3^ Later, geranyl diphosphate, a terpenoid prenyl group, is introduced by a prenyl transferase to generate cannabigerolic acid (CBGA), the progenitor of all other phytocannabinoid classes.^3^ In particular, tetrahydrocannabinolic acid (THCA) and cannabidiolic acid (CBDA) are both produced from CBGA by THCA synthase and CBDA synthase, respectively.^4^ These acidic forms of cannabinoids undergo nonenzymatic decarboxylation (typically through heat or aging), converting them into their neutral forms, which are capable of interacting with the receptors of the endocannabinoid system, thus exhibiting their biological activities. Tetrahydrocannabinol (THC) is a fairly potent agonist of the CB1 cannabinoid receptor,^5^ thus being responsible for the euphoriant intoxicating effect of cannabis, and also known for exerting significant pain-relieving activity.^6^ Cannabidiol (CBD), on the other hand, does not induce psychoactive effects, but has gained interest for its anti-inflammatory, anxiolytic, antipsychotic, and neuroprotective properties.^7^

Owing to the remarkable genetic plasticity of *C. sativa* and its adaptability to a wide range of pedoclimatic conditions, cultivators and breeders worldwide have long been developing an array of novel varieties.^8^ These include strains selectively enriched in specific phytocannabinoids, enhanced for increased cannabinoid yield, or tailored to exhibit distinctive and refined flavor profiles.^9^ For these reasons, a cannabis strain classification system based on the relative abundances of the three main phytocannabinoids has been proposed.^10–12^ Five distinct chemical phenotypes (chemotypes) have been described: type I plants, characterized by a predominance of THC; type II, with balanced abundance of THC and CBD; type III, defined by CBD as the dominant cannabinoid; type IV, in which CBG is the main phytocannabinoid; type V, with undetectable levels of phytocannabinoids.

Despite these efforts, the classification of *C. sativa* based on the sole major phytocannabinoids may be inadequate. Emerging research indicates that minor phytocannabinoids may play a more significant role than previously anticipated in influencing the pharmacological properties of cannabis.^13,14^ Notably, Berman et al. demonstrated that equally high-CBD extract from distinct cannabis accessions induced different anticonvulsant effects using the penylentetrazol test in mice, thus implying a synergistic effect of minor phytocannabinoids in the health benefits of cannabis.^15^ These phytocannabinoids are frequently referred to as “minor” due to their comparatively lower concentrations relative to the more predominant counterparts.^13^ Several of these identified and isolated molecules are structural homologues of THC and CBD with shorter or longer side chains on the phenolic moiety. Among them, the propyl homologues, termed varinoids, such as tetrahydrocannabivarin (THCV), have long been known and investigated for their biological properties.^16–18^ In 2019, tetrahydrocannabiphorol (THCP), the heptyl homologue of THC, was isolated and fully characterized for the first time alongside its CBD-type counterpart (CBDP).^19^ Remarkably, THCP demonstrated an *in vitro* affinity for the CB1 receptor more than 30 times greater than that of THC. Since then, several other minor compounds have been isolated, including the butyl^20,21^ and hexyl^22^ homologues. The growing diversity of these minor phytocannabinoids has necessitated the development of highly sensitive untargeted analytical methods to enable comprehensive phytocannabinoid profiling. High-resolution mass spectrometry (HRMS) has been emerging as the leading technology,^23^ and tools developed for metabolomics analyses have been adapted to phytocannabinoid profiling.^24–26^ In 2021, our research group introduced the term “phytocannabinomics” to describe this integrative approach.^27^ In the latter study, a data matrix comprising over 100 annotated phytocannabinoids revealed the existence of subgroups within the traditional classifications shaped by unique compositions of the minor phytocannabinoids. Notwithstanding, untargeted HRMS presents substantially greater analytical and interpretative challenges than conventional approaches.^28^ As such, metabolomics-like data sets can reach tens to hundreds of gigabytes, and preprocessing of the data through software programs is needed for data handling and compound annotation.^29^ In this study, Kendrick mass defect (KMD) filtering was employed for the first time in the untargeted annotation of phytocannabinoids. KMD filtering enables rapid visualization of compound homologues that differ by specific repeating molecular units (e.g., CH_2_)^30^ and has been widely applied in various analytical contexts, from environmental analysis^31^ to bioanalytics.^32^ In the present study, three different approaches for KMD filtering have been tested and compared by taking into account the phytocannabinoid coverage, false positive rates, computation burden, and versatility. This comparative evaluation demonstrates how KMD filtering advances beyond conventional HRMS approaches by streamlining data handling, reducing annotation complexity, and facilitating the detection of minor phytocannabinoids that might be overlooked in standard metabolomics approaches. The findings establish KMD filtering as a generalizable framework for comprehensive phytocannabinoid profiling and natural product discovery.

## 2 Materials and Methods

### 2.1 Phytocannabinoid Nomenclature

Compounds containing the typical pentyl-resorcinyl moiety retain their base name without any suffix, e.g., THC or CBD. Propyl analogues, the most common unorthodox alkyl chain homologues, have been named varinoids, since the *m*-propyl resorcinol is also known as divarinol. Therefore, propyl homologues are labeled by the suffix -varin (V), e.g., THCV and cannabidivarin (CBDV). Similarly, methyl, butyl, hexyl, and heptyl homologues have been given suffixes -orcol (O), -butol (B), -hexol (H), and -phorol (P), respectively. There is still no nomenclature for ethyl homologues; thus, they have been labeled as C2. Acid counterparts have been given the standard nomenclature for carboxylic acids (A); therefore, the acid form of THC is THCA. Finally, *O*-methyl compounds are labeled by a letter M, which precedes the eventual letter A, such as *O*-methyl cannabidiolic acid (CBDMA). Unless indicated otherwise, THC is the most abundant natural isomer (−)-*trans*-Δ^9^-THC.

### 2.2 Chemicals and Reagents

Analytical grade ethanol (96% yield) and LC-MS grade acetonitrile, water, and formic acid were purchased from Carlo Erba (Milan, Italy). Stock solutions of pure certified analytical standards of CBGA, CBDA, THCA, cannabigerovarinic acid (CBGVA), cannabidivarinic acid (CBDVA), tetrahydrocannabivarinic acid (THCVA), cannabichromevarinic acid (CBCVA), cannabichromenic acid (CBCA), and cannabinolic acid (CBNA), all in a 1 mg/mL methanol solution, were bought from Cerilliant (Sigma-Aldrich Merck, Milan, Italy). Cis-THCA, cannabidibutolic acid (CBDBA), tetrahydrocannabibutolic acid (THCBA), cannabihexolic acid (CBDHA), and cannabiphorolic acid (CBDPA) were obtained from previous syntheses.

### 2.3 Plant Material and Sample Preparation

Fifty cannabis genotypes were cultivated in the facilities of the Research Center for Cereal and Industrial Crops (CREA-CI) located in Rovigo, Italy, authorized under Article 26 of D.P.R. 309/90 for scientific purposes. Seeds were sown in March 2018, and harvesting was completed by November 2018. Mature female or monoecious inflorescences were collected at maturity and dried either by air (when feasible) or in an oven at 40 °C. Detailed characterization of each plant sample, i.e., plant material, sex, drying method, cultivation type, primary cannabinoid(s), chemotype, intended use, geographic origin, and designation of origin, is provided in Table S1.

Two grams of each accession were finely ground and subjected to extraction under the *Cannabis flos* monograph of the German Pharmacopeia as previously described.^25^ Specifically, 500 mg of the powdered material was suspended in 20 mL of 96% ethanol and stirred at room temperature for 15 min. The supernatant was collected, and the remaining residue was subjected to two additional extractions with 12.5 mL of 96% ethanol each. The resulting extracts were combined and brought to a final volume of 50 mL with 96% ethanol. Subsequently, 1 mL of the extract was filtered through a 0.45 μm cellulose membrane filter, and a 100 μL aliquot of the filtrate was diluted to a final volume of 1 mL using a mobile phase consisting of water and acetonitrile (30:70, *v*/*v*) containing 0.1% formic acid, yielding a final dilution factor of 10.

### 2.4 Untargeted HRMS Data Acquisition

Chromatographic separation was performed using an Ultimate 3000 UHPLC system (Thermo Fisher Scientific, Bremen, Germany) equipped with a vacuum degasser, binary pump, thermostated autosampler, and thermostated column compartment. The system was coupled to a Q-Exactive Orbitrap mass spectrometer (Thermo Fisher Scientific) via a heated electrospray ionization (HESI) source. Separation was achieved on a core–shell stationary phase reversed-phase (RP) column (Poroshell 120 SB-C18, 3.0 × 100 mm, 2.7 μm; Agilent, Milan, Italy), with mobile phases composed of H_2_O/HCOOH 99.9:0.1 (*v*/*v*, A) and ACN:HCOOH 99.9:0.1 (*v*/*v*, B). Chromatographic and spectrometric parameters were set as previously described.^25^ For MS, the parameters of the HESI source were set as follows: capillary temperature, 320 °C; vaporizer temperature, 280 °C; electrospray voltage, 3.8 kV; sheath gas, 55 arbitrary units (a.u.); auxiliary gas, 30 au; S lens RF level, 45.4. The acquisition software Xcalibur 3.0 (Thermo Fisher Scientific, San Jose, CA) was employed for full scan data-dependent acquisition (DDA) in negative ionization mode at a resolving power of 70,000 (full width half-maximum, fwhm, at *m*/*z* 200); scan range, *m*/*z* 150–750; automatic gain control (AGC) target, 3e06; injection time, 100 ms; isolation window, *m*/*z* 0.7. The sample volume injected for the analyses was 5 μL.

### 2.5 Phytocannabinoid Data Processing

The *raw* data sets of all samples and the process blank were preprocessed using the software Compound Discoverer version 3.1 (Thermo Fisher Scientific) using three distinct strategies: (i) KMD filtering before compound detection, (ii) KMD filtering after compound detection, and (iii) pseudo-KMD filtering using the expected compounds tool. The methods were applied to three macroclasses of cannabinoids based on their molecular weight and KMD, i.e., −0.172 (CBGA-type), −0.185 (THCA-type, CBDA-type, and CBCA-type), and −0.212 (CBNA-type and cannabinodiolic acid (CBNDA)-type). All data processing workflows aligned the features, detected the compound ions, grouped the different ions of the same molecules, and predicted the molecular formulas from MS1 and MS2 data. Moreover, the “fill gaps” tool was employed to find chromatographic peaks that were not detected by the “Detect Compounds” tool in all input files. For comparison, a standard metabolomics-based approach was tested and compared.

#### 2.5.1 KMD Filtering Before the “Compound Detection” Tool

In this approach, the “Filter By Mass Defect” tool was employed to filter out all peaks whose MS1 data lay outside the specified mass defect windows (Figure S1). The following parameters were applied: filter direction, keep; mass defect type, Kendrick mass defect; Kendrick formula, CH_2_; mass tolerance, 200 Da; and custom compositions, C_22_H_30_O_4_, C_22_H_32_O_4_, and C_22_H_26_O_4_. Different mass defect tolerance values were tested and compared: 0.025, 0.01, 0.005, 0.004, 0.0025, and 0.001. After the data processing, the following filters were applied: “MS2 is not equal to no MS2” (that removes all features whose MS1 data lack corresponding MS2 data) and “Formula is not blank” (that removes all features whose molecular formula could not be predicted).

#### 2.5.2 KMD Filtering after the “Compound Detection” Tool

In this approach, the “Calculate Mass Defect” tool was enabled after the “Group Compounds” tool (Figure S2). The following parameters were applied: Kendrick mass defect, true; Formula 1, CH_2_. After the data processing, the same filters reported in Section 2.5.1 were applied. In addition, the following filters based on KMD were applied using the “OR” mode, meaning that the features were kept if they fulfill at least one of the following: “Mass defect is between −0.184 and −0.186 in type KMD (CH_2_)”, “Mass defect is between −0.171 and −0.173 in type KMD (CH_2_)” or “Mass defect is between −0.211 and −0.213 in type KMD (CH_2_)”.

#### 2.5.3 Pseudo-KMD Filtering Using the “Expected Compounds” Tool

This approach mimicked KMD filtering, taking advantage of the “expected compounds” workflow (Figure S3). As such, the “Generate Expected Compounds” tool was enabled to generate the compound homologues. For this purpose, three compound progenitors were inserted, i.e., CBG­(C10)­A (C_27_H_42_O_4_, for the KMD – 0.172 macroclass), CBD­(C10)­A (C_27_H_40_O_4_, for the KMD – 0.185 macroclass), and CBN­(C10)­A (C_27_H_36_O_4_, for the KMD – 0.212 macroclass). Then, the demethylation phase I transformation was selected, and 10 maximum steps were considered. Then, the “Find Expected Compounds” tool was enabled before compound detection with the following parameters: mass tolerance, 5 ppm; intensity tolerance, 30%; intensity threshold, 0.1%; SN threshold, 3; minimum number of isotopes, 2; minimum peak intensity, 1000. To ensure proper alignment of the expected compound, we also enabled the “Group Expected Compounds” tool. Finally, the results from the expected compound annotation and compound detection were merged by using the “Merge Features” tool. After the data processing, the same filters reported in Section 2.5.1 were applied to the expected compounds.

### 2.6 Phytocannabinoid Annotation

MS/MS spectra of the filtered features were manually validated to assign the tentative identification according to the typical fragmentation pathways of phytocannabinoid classes.^15^ Data for the tentatively identified compounds are summarized in Table S2 with the related confidence level according to Schymanski et al.^33^ Specifically, level 1 identification denotes compounds unequivocally characterized through the concordance of exact mass, MS2, and RT with those of authentic reference standards, level 2 pertains to tentative identifications based on exact mass and MS2 spectra based on the known fragmentation rules of phytocannabinoid classes, and level 3 encompasses putative compounds for which the molecular formula and key functional groups are inferred, although full structural elucidation remains incomplete.

### 2.7 Statistical Analysis

Statistical analyses and data visualization were conducted using MetaboAnalyst 6.0.^34^ Following the guidelines provided by the developers, the data matrix was uploaded as a text file. Data filtering was performed using the interquartile range (IQR) method, while autoscaling was applied for data normalization. The annotated data matrices of the annotated phytocannabinoids were processed through MetaboAnalyst to generate hierarchical clustering outputs (dendrogram and heatmap), principal component analysis (PCA), partial least-squares-discriminant analysis (PLS-DA), and correlation heatmaps. Cannabis samples were grouped based on the geographical origin of the seeds, chemotype, and type of flower (monoecious vs dioecious).

## 3 Results and Discussion

### 3.1 Setup and Comparison of the KMD Filtering Strategy

The annotation of selected analytes, such as structurally related classes of compounds, from HRMS data remains a major challenge in untargeted metabolomics.^28^ Whereas the data acquisition strategies are well-established,^29^ significant challenges remain in processing and annotating the vast volumes of HRMS data. Unlike targeted methods, HRMS data sets can reach hundreds of GB, requiring software for peak detection, alignment, adduct annotation, formula prediction, and normalization.^35^ Once all of these operations are concluded, a list of thousands of peaks is obtained, and MS spectra annotation becomes the bottleneck of these studies. Identifying new metabolites and natural products is expensive, time-consuming, and labor-intensive.^36^ Although there is debate over how many detected features truly represent metabolites, it is widely believed that a large portion of compounds remains unknown and uncharacterized.^37,38^ When “minor” compounds belonging to structurally related classes, such as phytocannabinoids, are investigated, there is a need for data processing tools that allow streamlined analyses of complex data sets. Here, we present, for the first time, a comparative evaluation of Kendrick Mass Defect (KMD)-based filtering workflows for the annotation of minor phytocannabinoids using Compound Discoverer, a modular software platform for small molecule analysis based on customizable blocks and nodes. The core idea is to filter out peaks with KMD that are inconsistent with those of phytocannabinoids. Three distinct approaches for KMD filtering were tested and compared: (i) KMD filtering before the “Compound Detection” tool (KMD before CD), (ii) KMD filtering after the “Compound Detection” tool (KMD after CD), and (iii) pseudo-KMD filtering using the “Expected Compounds” tool (pseudo-KMD with EC). To evaluate the workflows in terms of phytocannabinoid coverage, false positive rates, computation burden, and versatility, 50 cannabis flowers were extracted and analyzed by untargeted HRMS in negative ion mode (around 6 GB of data). The plant material was not subjected to decarboxylation; thus, the acidic forms of phytocannabinoids were taken into consideration. Based on their KMD, three macroclasses of phytocannabinoids were investigated: −0.172 (CBGA-type), −0.185 (THCA-type, CBDA-type, CBCA-type, CBLA-type, and sesquiCBGA-type), and −0.212 (CBNA-type and CBNDA-type). As a reference, a conventional untargeted metabolomics workflow was applied, yielding 2868 filtered features and 62 annotated cannabinoids after careful manual MS spectra inspection, corresponding to an annotation rate (annotated features/filtered features ratio) of 2.2% (Table 1).

**Table 1:** Summary of the Performance of KMD before CD, KMD after CD, and Pseudo-KMD with EC for the Annotation of Phytocannabinoids from Untargeted HRMS Data

	KMD tolerance	filtered features	annotated cannabinoids	annotation rate	storage size
No KMD	-	2868	62	2.2%	6.0 GB
KMD before CD	0.025	1208	62	5.1%	1.7 GB
KMD before CD	0.01	817	61	7.5%	1.1 GB
KMD before CD	0.005	410	61	14.9%	0.8 GB
KMD before CD	0.004	292	37	12.7%	0.6 GB
KMD before CD	0.0025	40	2	5.0%	0.5 GB
KMD before CD	0.001	0	0	-	0.3 GB
KMD after CD	-	288	61	21.2%	6.0 GB
Pseudo-KMD with EC	-	414	59	14.3%	6.2 GB
KMD before+after CD	0.005	287	61	21.3%	0.8 GB

#### 3.1.1 KMD Filtering before the “Compound Detection” Tool

The KMD before CD approach is based on the addition of the “Filter by Mass Defect” block between “Align Retention Times” and “Detect Compounds” (Figure S1). Once the spectra are selected and aligned, an automated filter is applied to the list of extracted *m*/*z* values to remove all peaks that lie outside a specific KMD tolerance. As a result, all subsequent operations, including time-consuming “Detect Compounds”, “Fill Gaps”, and “Predict Composition”, are carried out on a significantly reduced list of peaks. Thus, KMD, before CD, significantly reduces the duration of the processing operations and the processed file size. KMD tolerance is the key parameter to optimize in order to achieve effective filtering while preserving analytes of interest. Six different values have been tested and compared, i.e., 0.025 (the default value), 0.01, 0.005, 0.004, 0.0025, and 0.001. The results are summarized in Table 1. Storage size was used as a proxy for computational burden since processing time may vary with hardware performance and background tasks. At KMD tolerance 0.025, the filtered features were 1208, a 58% reduction compared to the standard workflow, resulting in a substantial reduction in file size to 1.7 GB. Lowering the KMD tolerance to 0.01 and 0.005 had a significant effect on the reduction of the filtered features without compromising the annotated phytocannabinoids. In particular, at 0.005 tolerance, the number of filtered features was reduced to 410, corresponding to an 86% decrease and an annotation rate of almost 15%. Nevertheless, a further reduction of the KMD tolerance led to the removal of several phytocannabinoids from the data sets, indicating that KMD tolerances below 0.005 approach the mass accuracy limits of the HRMS data. Reducing the tolerance to 0.004 led to a significant loss of annotations (only 37 compounds were identified), and the annotation rate fell to 12%. At 0.001, none of the peaks in the data sets were kept. For these reasons, the KMD tolerance should be optimized for each data set, as a slight decrease (from 0.005 to 0.004) had a significant repercussion on the number of annotated compounds.

#### 3.1.2 KMD Filtering after the “Compound Detection” Tool

The KMD after CD approach is based on the use of the “Calculate Mass Defect” tool after compound detection and grouping (Figure S2). This tool does not automatically remove peaks but rather calculates the KMD (once the parameters are adapted to a specific kind of mass defect) of all peaks extracted from the data sets. Therefore, the computational burden is not reduced, but there is no need to worry about the potential unwanted removal of analytes of interest. Following the completion of the data analysis, additional filters based on the KMD were added, as described in Materials and Methods. As a result, compared to the standard metabolomics workflow, around 9 out of 10 features are filtered out (− 90%). Despite this, the number of annotated phytocannabinoids mirrored that of the KMD before CD workflow, resulting in an annotation rate of almost 21%.

#### 3.1.3 Pseudo-KMD Filtering Using the “Expected Compounds” Tool

In the pseudo-KMD with the EC workflow, there is no real filtering from the list of extracted features (hence pseudo-KMD). Rather, a customized “Expected Compounds” workflow was set up to obtain a list of minor phytocannabinoid candidates that mimics the KMD filtering approach. The “Expected Compounds” tool is typically used to search for drug metabolites based on simulated degradation reactions.^39^ As shown in Figure S3, four new blocks were added to the workflow. The “Generate Expected Compounds” tool generates the expected compounds based on the starting compounds and the chosen reactions. In this workflow, CBDA, CBGA, and CBNA homologues with 10 carbon atoms in their side chain were selected as starting compounds. As for the reactions, the sole demethylation reaction was selected, and a maximum of 10 reactions were considered. This compound generation approach effectively searches for all homologues, thereby simulating the effect of KMD filtering. The “Find Expected Compounds” and “Group Expected Compounds” tools are connected to the “Align Retention Times” block to search and group the expected compounds from the aligned peak list. Finally, the “Merge Features” tool combined the expected compounds and the unknown compounds found by the Detect Compounds node. This workflow generates a list of expected compounds that were filtered to remove all peaks that were not associated with an MS/MS spectrum. The workflow’s complexity increased the computational burden, only partially reflected by the slightly larger file size (6.2 vs 6.0 GB). A total of 414 expected compounds were obtained, resulting in 59 annotations (annotation rate of 14%). The relatively higher number of filtered features is likely due to the impossibility, using the 3.1 version of Compound Discoverer, of enabling the gap filling of the expected compounds, which would result in much better alignment, especially for low-abundance compounds.

#### 3.1.4 Comparison of the Workflows and Choice of the Final Method

Figure 1 displays the Kendrick diagrams using the standard metabolomics workflow (a), the KMD before CD workflow at 0.005 tolerance (b), the KMD after CD workflow (c), and the pseudo-KMD with the EC workflow (d). Filtered peaks are marked in blue, whereas annotated phytocannabinoids are marked in red. The reduction of the filtered features appears evident by comparing the standard approach to the three KMD filtering workflows. Each graph shows fewer dots than the number of features due to overlapping isomers in the Kendrick diagrams. This is particularly significant for the pseudo-KMD with EC, with 414 features and 29 dots. In this case, other than the numerous isomers, there is the poor alignment of some low-abundance nonidentified peaks. In terms of compound coverage, the three methods were comparable, with 61, 61, and 59 annotated phytocannabinoids, respectively, meaning that only 1 to 3 compounds were erroneously filtered out. Among these, an unknown CBDA isomer (*m*/*z* 357.2078 and RT 17.81) was recurring among the three approaches based on the wrong prediction of the molecular formula (and therefore KMD).

**Figure 1:**
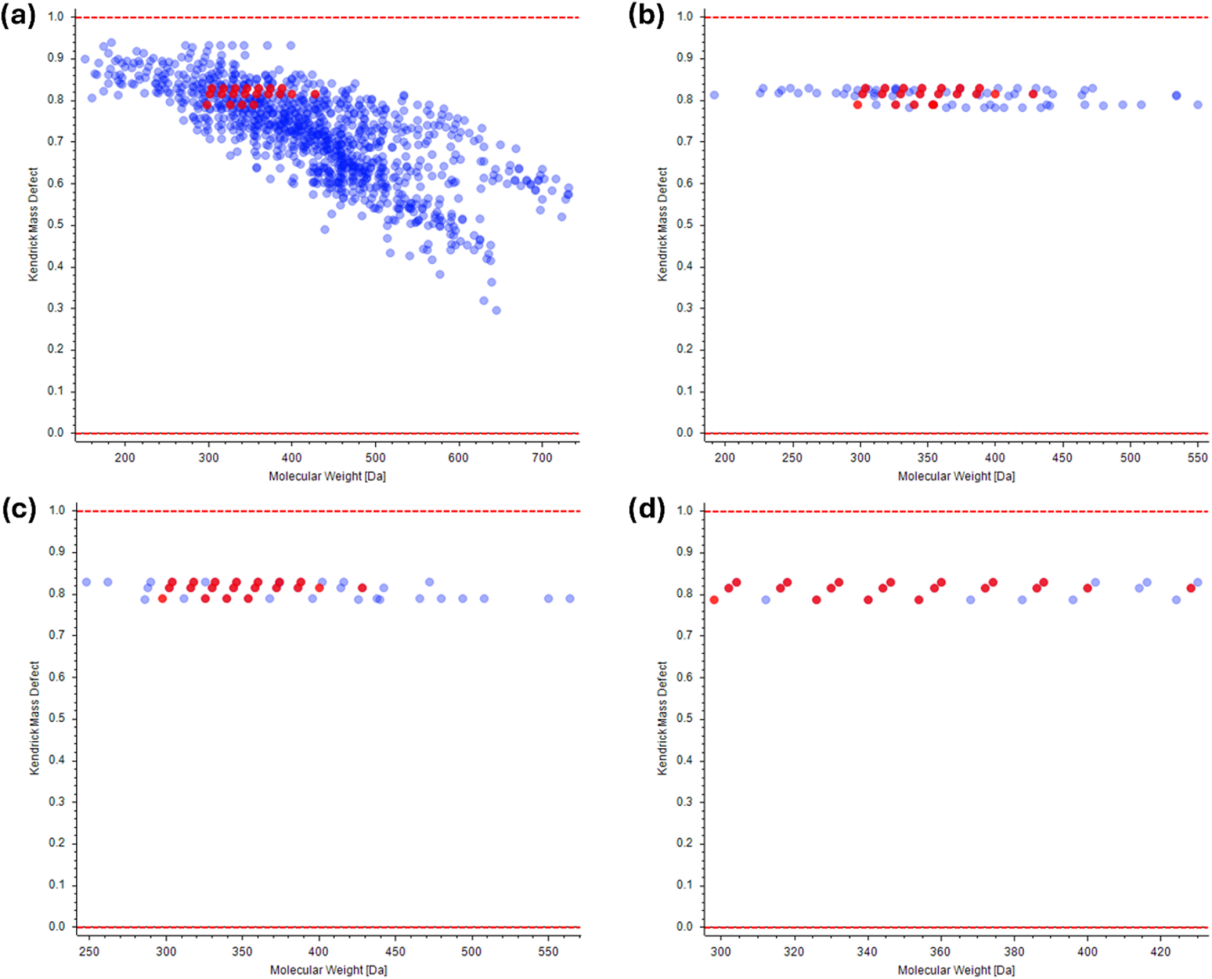
Kendrick diagrams of the filtered features following the data processing workflow of the phytocannabinoid data set using (a) a standard metabolomics-based workflow, (b) KMD filtering before the “Compound Detection” tool, (c) KMD filtering after the “Compound Detection” tool, and (d) pseudo-KMD filtering using the “Expected Compounds” tool. Annotated phytocannabinoids are marked in red.

Each workflow showed specific strengths and weaknesses. KMD before CD effectively streamlines analysis by reducing the computational load, making it ideal for high-throughput workflows. Despite this advantage, this approach is the least versatile. First, once the processing is concluded, the discarded features cannot be retrospectively analyzed. Moreover, up to 5 compound classes can be inserted into the “Filter By Mass Defect” tool, posing a limitation on how many classes can be analyzed at once. The KMD after CD is the simplest approach to set up, as there is only an additional step added to the standard workflow. Therefore, other compound classes can be analyzed retrospectively based on other KMD filtering. Despite being incapable of reducing the computational burden, it reached the highest annotation rate. As regards the pseudo-KMD with EC, the results in Table 1 might appear the least satisfactory, with the highest storage size, the lowest annotation rate, and the lowest phytocannabinoid coverage. The most significant issue is related to the incompatibility with the “Fill Gaps” tool, which not only increased the number of features but also resulted in a data matrix in which peaks not found in certain samples were left with a peak area equal to zero. On the other hand, the gap-filling procedure ensures that all peaks are correctly aligned, substitutes missing peaks with the noise level, and allows quality control correction mode. However, the “Find Expected Compounds” approach has the highest potential in investigating other phytocannabinoid structures based on unreported modifications. As such, this approach represents a powerful tool in exploring the full diversity of phytocannabinoids, whereas the other two methods are more helpful in simplifying the annotation of phytocannabinoids with known or suspected structures.

Table 2 summarizes the pros and cons of each of the tested approaches. To address the limitations of the individual approaches, we tested a fourth strategy, i.e., KMD before+after CD workflow, that combined the two blocks employed in the first two tested methods. This hybrid method capitalized on the strengths of the first two methods, i.e., it reduced the computational burden as in KMD before CD while retaining the higher annotation rate of KMD after CD. With the aim of streamlining HRMS-based untargeted phytocannabinoid analysis, this combined approach achieved the best balance across performance metrics, with reduced computational requirements, robust phytocannabinoid coverage (61 annotations), and the highest overall annotation efficiency. However, this approach inherited the lack of versatility of the KMD before the CD workflow. Thus, while this fourth approach is the optimum for streamlined and robust phytocannabinoid homologue annotation, more versatile methods would be preferable for unknown compound discovery

**Table 2:** Summary of the Pros and Cons of the Three KMD Filtering Approaches for Minor Phytocannabinoid Untargeted Analysis

method	pros	cons
KMD before CD	• significant reduction of the computational burden and storage size	• KMD tolerance may lead to filtering out compounds of interest
	• KMD tolerance can be optimized	• suboptimal reduction of the filtered features
KMD after CD	• maximum reduction of the filtered features	• no reduction of the computational burden and storage size
	• other compound classes can be filtered from the data sets	
pseudo-KMD with EC	• other compound classes can be added to the method	• incompatibility of the “Fill Gaps” tool
	• other modifications of the structures can be added to the method	• suboptimal reduction of the filtered features
		• higher computational burden and storage size

### 3.2 Composition of the Annotated Physicocannabinoids in Cannabis Inflorescence

As previously described, a total of 61 phytocannabinoids were annotated using the KMD before+after CD approach, 14 of which were confirmed by matching the experimental data to the RT, MS, and MS/MS of available standards. THCA-type phytocannabinoids were the most numerous, including the full series (1–7 carbon atoms in the side chain) of the major trans-Δ^9^ isomer, five cis-Δ^9^ isomers (the complete series except the C2 and C7 homologues), and two trans-Δ^8^ isomers (Δ^8^-THCOA and Δ^8^-THCA). Several unknown THCA and THCVA isomers were also detected, in line with previous findings on annotated phytocannabinoids from isolated trichomes.^40^ Similarly, the complete series of CBDA-, CBGA-, and CBCA-type phytocannabinoids were annotated together with cannabicyclolic acid (CBLA) and cannabicyclovarinic acid (CBLVA). In addition, six O-methylated phytocannabinoids were annotated, belonging to the CBDA- and CBGA-type classes. Eight CBNA/CBNDA-type compounds were identified, including two unknown isomers of CBNA and CBNDA. Finally, sesquiCBGA and sesquiCBGVA, homologues of CBGA and CBGVA with a longer prenyl chain, were also tentatively identified since their mass composition corresponds to the KMD – 0.185 macroclass.

While CBGA-type cannabinoids are distinct in mass composition, THCA-, CBDA-, CBCA-, and CBLA-type phytocannabinoids are structural isomers, i.e., they all belong to the macroclass characterized by KMD −0.185. Differentiation was achieved through a combination of MS/MS fragmentation patterns and retention behavior in RP chromatography. CBDA-type compounds, with two free hydroxyl groups, showed significantly earlier elution than monohydroxylated THCA-, CBCA-, and CBLA-types (typical RT order: CBDA ≪ THCA < CBCA < CBLA). The longer free prenyl side chains of CBCA and CBLA contribute to increased hydrophobicity and retention. In terms of the MS/MS spectra, THCA-type compounds, with an extensive conjugated and planar structure, are much more poorly fragmented compared to the other isomers. Moreover, CBDA- and CBCA-types are easily distinguished by the ions produced by the cleavage of the prenyl moiety, i.e., *m*/*z* 245.1547 and 243.1386, respectively.^15^

The analysis of the RT was also considered for homologue annotation, as each extra methylene was expected to increase the RT in RP separation, e.g., CBDBA (*m*/*z* 343.1919, RT = 16.7) < CBDA (*m*/*z* 357.2076, RT = 17.4) < CBDHA (*m*/*z* 371.2232, RT = 18.1). In the case of *O*-methylated compounds, the contribution of this methylene to the compound hydrophobicity is much more significant than that of a longer alkyl chain; therefore, *O*-methyl phytocannabinoids are much more retained than the longer alkyl chain isomers, e.g., CBDMA (*m*/*z* 371.2231, RT = 20.1) > CBDHA (*m*/*z* 371.2232, RT = 18.1). Following compound annotation, a data matrix of each compound’s content in the 50 analyzed samples was used for statistical analysis on Metaboanalyst. Four low-abundance compounds (CBC­(C2)­A, CBG­(C2)­A, cis-THCHA, and sesquiCBGVA) were excluded by interquartile range filtering, resulting in a final data set of 57 phytocannabinoids.

#### 3.2.1 Geographical Origin of the Seeds

The effect of the geographical origin of the plant material on the expression of phytocannabinoids was considered, i.e., European versus Chinese seeds (a single sample of American origin was not employed in these analyses). Figure 2a shows the resulting Volcano plot analysis (*t-*test vs fold-change). Despite uneven content in the major phytocannabinoids, there were several phytocannabinoids with consistently higher abundances in the samples of Chinese origin. In particular, all six annotated *O*-methyl phytocannabinoids were overexpressed in the latter.

**Figure 2:**
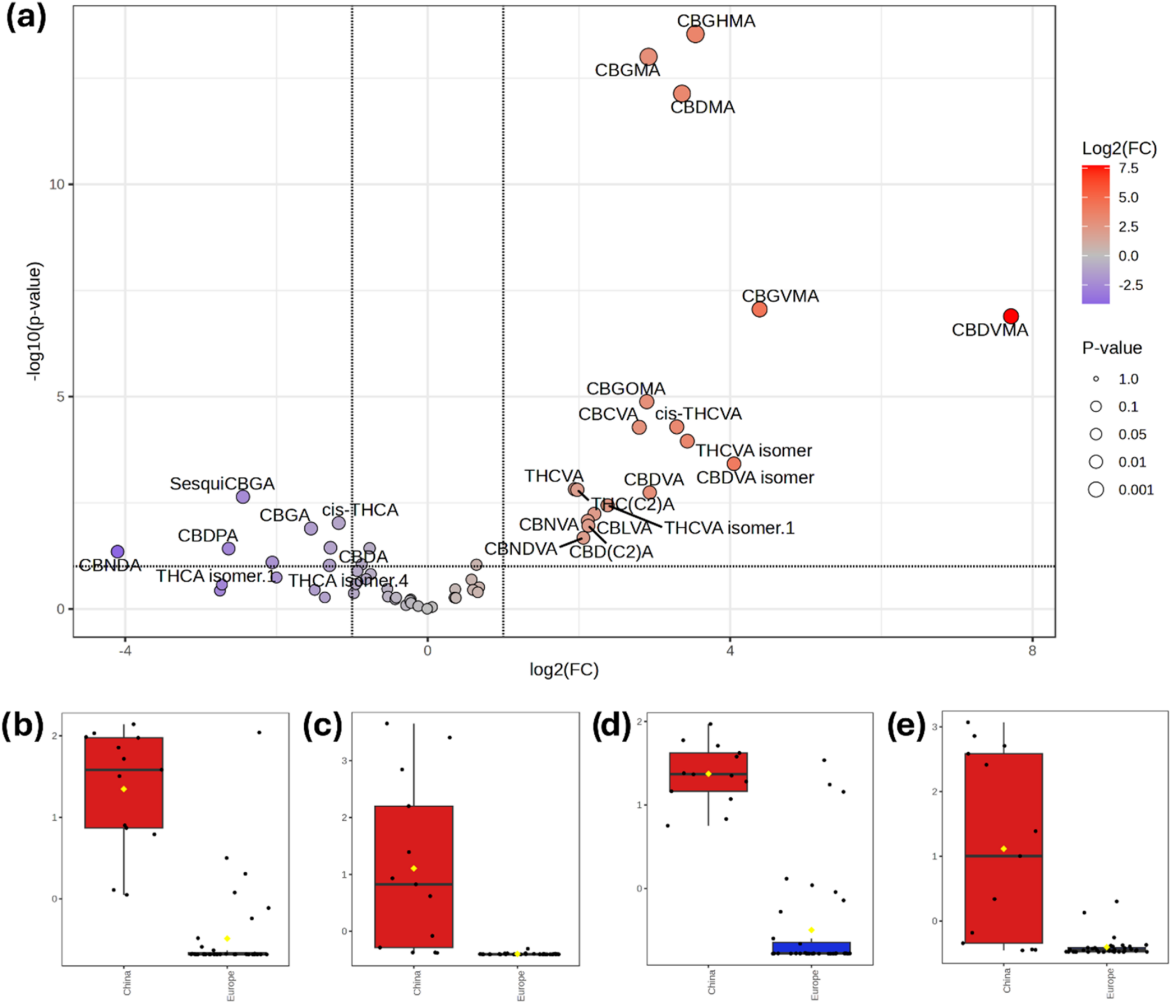
Volcano plot analysis using the phytocannabinoids data matrix comparing European vs Chinese cannabis varieties (a). Box and whisker plots of the peak areas of CBDMA (b), CBDVMA (c), CBGMA (d), and CBGVMA (e) in European and Chinese cannabis varieties.

Figure 2b–e shows the box-and-whiskers plots of CBDMA, CBDVMA, CBGMA, and CBGVMA, respectively, displaying the consistently higher abundances of these compounds in cannabis inflorescences obtained from the Far East. Despite having been first isolated by Shoyama et al. in the early 70s,^41,42^ little is known about the biological profile of *O*-methyl phytocannabinoids or the enzymes involved in their biosynthesis, even though Caprioglio et al. demonstrated that the *O*-methylation does not affect the peroxisome proliferator-activated receptors (PPARs), in contrast to what has been reported for phenethyl analogues.^43^ On the other hand, previous reports highlighted Far Eastern cannabis varieties enriched in *O*-methyl phytocannabinoids,^14^ and these compounds might serve as markers of the plant’s geographical origin regardless of the site of the cultivation. Univariate hierarchical clustering analysis (Figure S4) and multivariate PLS-DA (Figure S5a) demonstrated good classification of the samples, and variable importance analysis in projection (VIP) scores confirmed the overexpression of *O*-methyl phytocannabinoids (Figure S5b).

#### 3.2.2 Chemovar

Based on the standard classification, the 50 samples were grouped in chemovars: chemovar I (17 samples), chemovar II (6), chemovar III (19), chemovar IV (7), and chemovar V (1). For subsequent statistical analysis, the sole chemovar V sample was discarded. PCA of the phytocannabinoid data for the remaining four chemovars (explained variance of PC1+PC2 44.0%, Figure S6) revealed no distinct separation among groups, consistent with previous findings.^27^ In particular, there was clear overlapping of chemovars III and IV and partial overlapping of chemovars II and I, with the latter being significantly dispersed in the score plot. Box-and-whisker plots (Figure S7) for the main phytocannabinoids, THCA, CBDA, CBGA, and CBCA showed expected trends: THCA was most abundant in chemovars I and II; CBDA dominated chemovars II and III; and CBGA peaked in chemovar IV. Interestingly, CBCA levels were consistent across chemovars I and III but significantly lower in chemovar IV, suggesting that its biosynthesis may be equally linked to both THCA and CBDA pathways. The homologue series of THCA-, CBDA-, and CBGA-type compounds (Figures S8–S10) revealed broadly similar trends across chemovars. Although THCA-type homologues were consistently more abundant in chemovars I and II, CBDA- and CBGA-type homologues showed more variable patterns, particularly among long-chain derivatives (e.g., CBDHA, Figure S9f; CBGPA, Figure S10f). Thus, it can be hypothesized that, when all phytocannabinoids are taken into consideration, a classification of the cannabis samples in chemovars I+II and III+IV would be more effective. PCA and hierarchical clustering (Figure S11) of these “macrochemovars” provided clearer classification, and Volcano Plot analysis identified numerous phytocannabinoids with increased abundance in the I+II group. These included THCA-type and CBNA-type compounds, the latter being known for originating from oxidative reactions of THCA.^44^

However, given how dispersed the samples from chemovar I in the PCA were, further investigations were carried out. As such, if a subset of samples is considered (chemovar I only) and the geographical origin of the seeds (Europe vs China) is considered, then the results were in line with the previous findings described in paragraph 3.2.1. The two subgroups were clearly separated in the score plot of the PCA (Figure S12a), and the Volcano plot analysis confirmed the overexpression of *O*-methyl phytocannabinoids in the Far Eastern samples (Figure S12b). Based on these considerations, the sample classification based on the chemovar was rerun after removing all samples of Far Eastern origin. Interestingly, the PCA shows clear separation of chemovars I and II and much improved separation of chemovars III and IV (explained variance of PC1+PC2 = 45.7%, Figure S13). These results demonstrate that the geographic origin of the seeds is a confounding factor when classifying cannabis samples based on the chemovar, especially between chemovar I vs II and chemovar III vs IV. Overall, while chemovar-based classification provides a useful framework, the statistical evidence indicates that phytocannabinoid variability is only partly explained by the current chemovar categories, highlighting the need for refined classification schemes (e.g., macrochemovars) and careful control of confounding factors.

#### 3.2.3 Subchemovar

The investigation of possible subgroups within the existing chemovar was thoroughly investigated in our previous work.^27^ Although the main cannabinoid content was similar, the hypothesized subgroups differed in minor phytocannabinoid profiles, especially in terms of the C3 phytocannabinoids. The THCA/THCVA and C5/C3 (in which C5 = THCA + CBDA + CBGA and C3 = THCVA + CBDVA + CBGVA) ratios were therefore calculated for the samples belonging to chemovar I. Based on these ratios, two subgroups can be described: a more numerous group with both calculated ratios much higher than 1 (THCA ≫ THCVA, samples UN1, UN3–5, UN8–14), and a smaller group with ratios around 1 (THCA ≈ THCVA, samples UN2, UN6–7, UN15–17). The volcano plot analysis (Figure S14) demonstrated that the smaller subgroup had an increased abundance of low-carbon chain homologues (mainly C3 and C2) regardless of the phytocannabinoid class. These subgroups comprised genotypes of both European and Far Eastern origin, suggesting that the subgrouping is independent of geographic origin, at least within the chemovar I cohort. The correlation heatmap (Figure 3) built on this data set displays a highly correlated subset of phytocannabinoids of all classes with C3 and C2 side chains.

**Figure 3:**
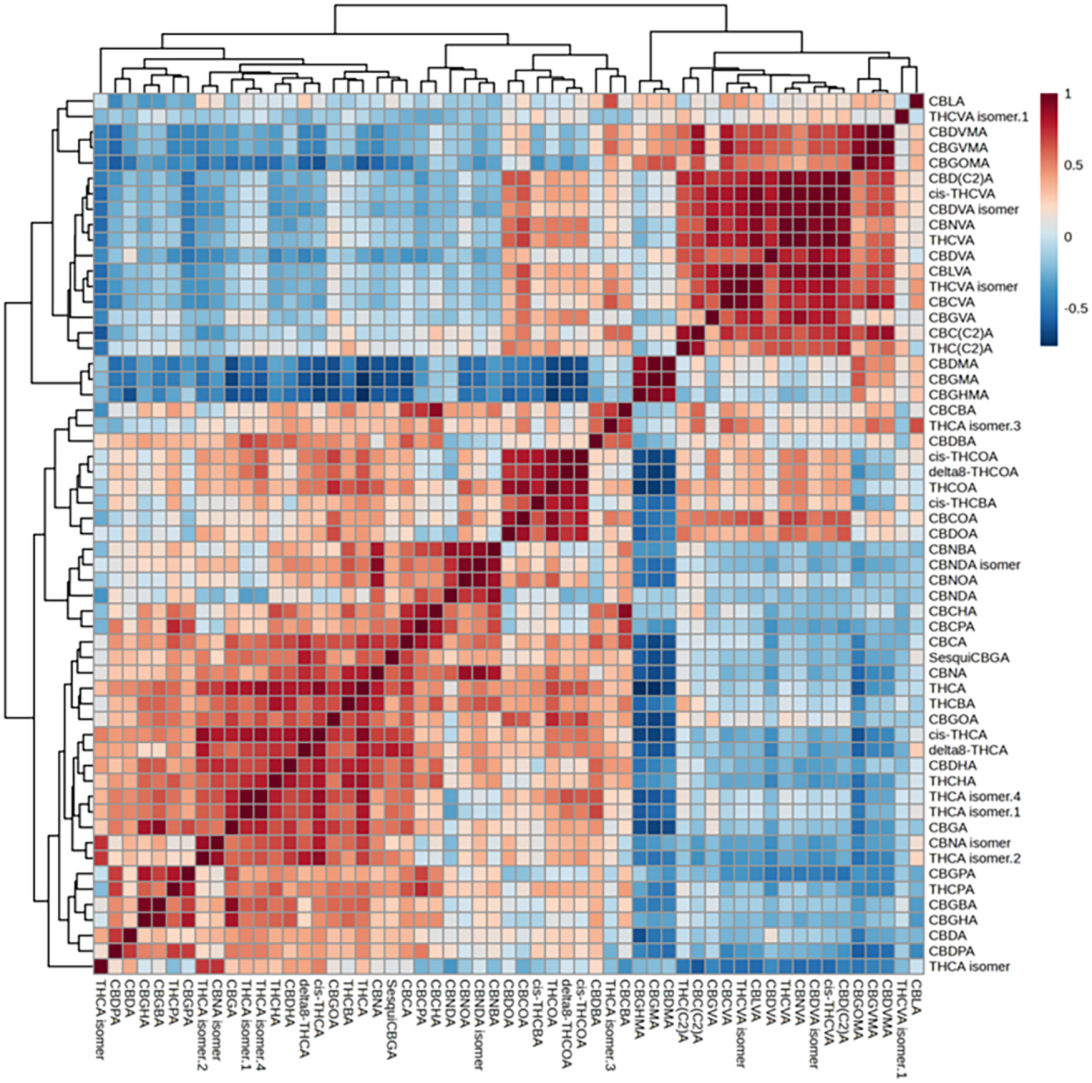
Correlation heatmap built using the phytocannabinoid data sets in the two defined subgroups of samples within chemovar I.

A larger subset of longer chain phytocannabinoids (C4–7) was also characterized by high Pearson correlation coefficients, whereas most C1 phytocannabinoids (known as orcinoids) correlated broadly across chain lengths, meaning that their synthetic pathways were likely not influenced by the differential enzymatic activity linked to the biosynthesis of C3 and C5 phytocannabinoids. Similar investigations were undertaken within the other subgroups. However, the C3-enriched subgroups of samples were either too small or too highly correlated with the geographical origin. Larger sets of samples would be needed to thoroughly investigate the matter.

#### 3.2.4 Plant Reproductive Strategy

Cannabis is usually considered a dioecious plant; i.e., each plant is either male or female, the former producing pollen and the latter producing seeds. Female plants are generally preferred for cultivation when the goal is the extraction of active compounds or use for consumption, as they produce in the flowers higher concentrations of the desired phytochemicals compared to male plants.^45^ However, some monoecious cannabis strains, bearing both male and female flowers on the same plant, have been developed by breeders^46^ especially for industrial application (i.e., seed hemp or seed/fiber hemp). The monoecious genotypes used in this work, as shown in Table S1, all belonged to chemovars III and IV. Therefore, cannabis samples from chemovars III and IV were grouped into dioecious and monoecious classes, and the phytocannabinoid data sets were subjected to statistical analysis. The volcano plot shown in Figure S15 displays a clear differential phytocannabinoid content between the two classes, with dioecious female plants producing significantly higher concentrations of phytocannabinoids than monoecious ones. As such, around half of the annotated phytocannabinoids were significantly overexpressed in the female inflorescences of dioecious strains, whereas none were overexpressed in the monoecious group. Among the overexpressed phytocannabinoids, there were both THCA and CBDA (but not CBGA), as well as several minor C1, C4, C6, and C7 homologues. Some examples are shown in the box-and-whiskers plots in Figure S15b–d. Given the comparable CBGA content, differential enzymatic activity may be hypothesized, possibly reflecting differences in the activity or expression of downstream synthase enzymes such as THCA or CBDA synthases within strains of nonpsychoactive cannabis.

## 4 Conclusion

This study presents the first comprehensive evaluation of KMD-based filtering strategies for the untargeted phytocannabinoid annotation in HRMS data sets. By leveraging the modular flexibility of Compound Discoverer, three distinct workflows were implemented and compared (alone and in combination), and the potential of KMD filtering was evaluated in terms of phytocannabinoid coverage, false positive rates, computational burden, and versatility. The integration of KMD filtering into the annotation workflow not only reduces the computational burden and data complexity inherent to untargeted HRMS but also enables a more targeted and chemically meaningful exploration of homologous compound families. The annotated phytocannabinoid profiles encompassed a broad spectrum of structural diversity, underscoring the cannabis chemical complexity. Importantly, the comprehensive annotation facilitated statistically meaningful comparisons across biological and agronomic categories. The results highlighted nuanced differentiations driven by seed geographic origin and reproductive strategy. Moreover, our results confirmed the hypothesis that current cannabis classification systems, often based solely on major cannabinoids, are insufficient to capture the full chemical heterogeneity of the plant. As demonstrated here, in-depth analysis of minor phytocannabinoids reveals chemovar substructures and biosynthetic nuances that are otherwise obscured in conventional classification schemes.

Looking forward, the integration of KMD filtering into untargeted workflows holds promise not only for phytocannabinomics but also for the broader fields of metabolomics and natural product discovery. Because KMD filtering highlights recurring structural patterns, it is well-suited to systematically detect and organize diverse classes of metabolites beyond phytocannabinoids, such as lipids, polyhydroxylated and polymethylated flavonoids, glycosides, and other compound families with repeating units. This makes it a broadly applicable strategy for prioritizing features in complex data sets, guiding annotation efforts, and uncovering novel analogues within natural products. Future studies should focus on expanding the library of known and putative phytocannabinoids, incorporating additional transformations into the expected compounds’ framework.

## Supplementary Material



## Notes

Details on the 50 analyzed *C. sativa* flower samples (Table S1); scheme of the three KMD filtering workflows on Compound Discoverer (Figures S1–S3); list of the 61 annotated phytocannabinoids (Table S2); hierarchical clustering heatmaps and dendrograms, PCA, PLS-DA, VIP analysis; box-and-whiskers plots; and volcano plots resulting from statistical analysis (Figures S4–S15); and THCA/THCVA and C5/C3 ratios from samples of chemovar I (Table S3) (PDF)
