# Supplementary material for: Kendrick Mass Defect Filtering Enables High-Throughput Untargeted Annotation of Minor Phytocannabinoids: Toward Streamlined Phytocannabinomics
Source: ACS Meas Sci Au. 2025 Oct 23;5(6):912–22. doi: 10.1021/acsmeasuresciau.5c00106 (PMC12715730; doi:10.1021/acsmeasuresciau.5c00106)
Supplement: Supplementary file 1 [file tg5c00106_si_001.pdf]

Supporting Information

Kendrick Mass Defect filtering enables high-throughput untargeted annotation of minor phytocannabinoids: toward streamlined phytocannabinomics

Andrea Cerrato,[1,2] Giuseppe Cannazza,[3,4] Cinzia Citti,[3,4] Aldo Laganà,[1,2] Roberta Paris,[5] Anna Laura Capriotti[1,2,*]

[1] Department of Chemistry, Sapienza University of Rome, Piazzale Aldo Moro 5, 00185, Rome, Italy

[2] Interuniversity Consortium INBB – Biostructures and Biosystems National Institute, Via dei Carpegna 19, 00165, Rome, Italy

[3] Department of Life Sciences, University of Modena and Reggio Emilia, Via Giuseppe Campi 287, 41125, Modena, Italy

[4] CNR NANOTEC, Campus Ecotekne, University of Salento, Via Monteroni, 73100, Lecce, Italy

[5] CREA, Research Centre for Cereal and Industrial Crops, Via di Corticella 133, Bologna, 40128, Italy

*Corresponding author

E-mail: annalaura.capriotti@uniroma1.it

tel: +39 06 4991 3945

Table S1. Details on the 50 Cannabis sativa flower samples analyzed in this study.

Sample_ID	Sex	Cultivation type	Chemotype	Use	Geographical origin
**UN1**	female	outdoor	I	Industrial	China
**UN2**	female	outdoor	I	Industrial	China
**UN3**	female	outdoor	I	Industrial	China
**UN4**	female	outdoor	I	Industrial	China
**UN5**	female	outdoor	I	Industrial	China
**UN6**	female	outdoor	I	Industrial	China
**UN7**	female	indoor	I	Pharmaceutical	Europe
**UN8**	female	indoor	I	Pharmaceutical	Europe
**UN9**	female	indoor	I	Pharmaceutical	Europe
**UN10**	female	indoor	I	Pharmaceutical	America
**UN11**	female	indoor	I	Pharmaceutical	Europe
**UN12**	female	indoor	I	Pharmaceutical	Europe
**UN13**	female	indoor	I	Pharmaceutical	Europe
**UN14**	female	indoor	I	Pharmaceutical	Europe
**UN15**	female	outdoor	I	Industrial	China
**UN16**	female	outdoor	I	Industrial	China
**UN17**	female	outdoor	I	Pharmaceutical	Europe
**UN18**	female	indoor	II	Pharmaceutical	Europe
**UN19**	female	indoor	II	Pharmaceutical	Europe
**UN20**	female	indoor	II	Pharmaceutical	Europe
**UN21**	female	outdoor	II	Industrial	China
**UN22**	female	outdoor	II	Industrial	China
**UN23**	female	outdoor	II	Industrial	China
**UN24**	monoecious	outdoor	III	Industrial	Europe
**UN25**	monoecious	outdoor	III	Industrial	Europe
**UN26**	monoecious	outdoor	III	Industrial	Europe
**UN27**	monoecious	outdoor	III	Industrial	Europe
**UN28**	monoecious	outdoor	III	Industrial	Europe
**UN29**	female	outdoor	III	Industrial	Europe
**UN30**	female	outdoor	III	Industrial	Europe
**UN31**	female	outdoor	III	Industrial	Europe
**UN32**	female	greenhouse	III	Industrial	Europe
**UN33**	female	outdoor	III	Industrial	Europe
**UN34**	female	outdoor	III	Industrial	Europe
**UN35**	female	outdoor	III	Pharmaceutical	Europe
**UN36**	female	outdoor	III	Pharmaceutical	Europe
**UN37**	monoecious	greenhouse	III	Industrial	Europe
**UN38**	female	outdoor	III	Industrial	Europe
**UN39**	female	outdoor	III	Industrial	Europe
**UN40**	female	indoor	III	Pharmaceutical	Europe
**UN41**	female	outdoor	III	Industrial	China
**UN42**	female	outdoor	III	Industrial	China

**UN43**	monoecious	outdoor	IV	Industrial	Europe
**UN44**	monoecious	outdoor	IV	Industrial	Europe
**UN45**	monoecious	outdoor	IV	Industrial	Europe
**UN46**	female	outdoor	IV	Industrial	Europe
**UN47**	monoecious	outdoor	IV	Industrial	Europe
**UN48**	female	outdoor	IV	Industrial	Europe
**UN49**	female	outdoor	IV	Industrial	Europe
**UN50**	monoecious	outdoor	V	Industrial	Europe

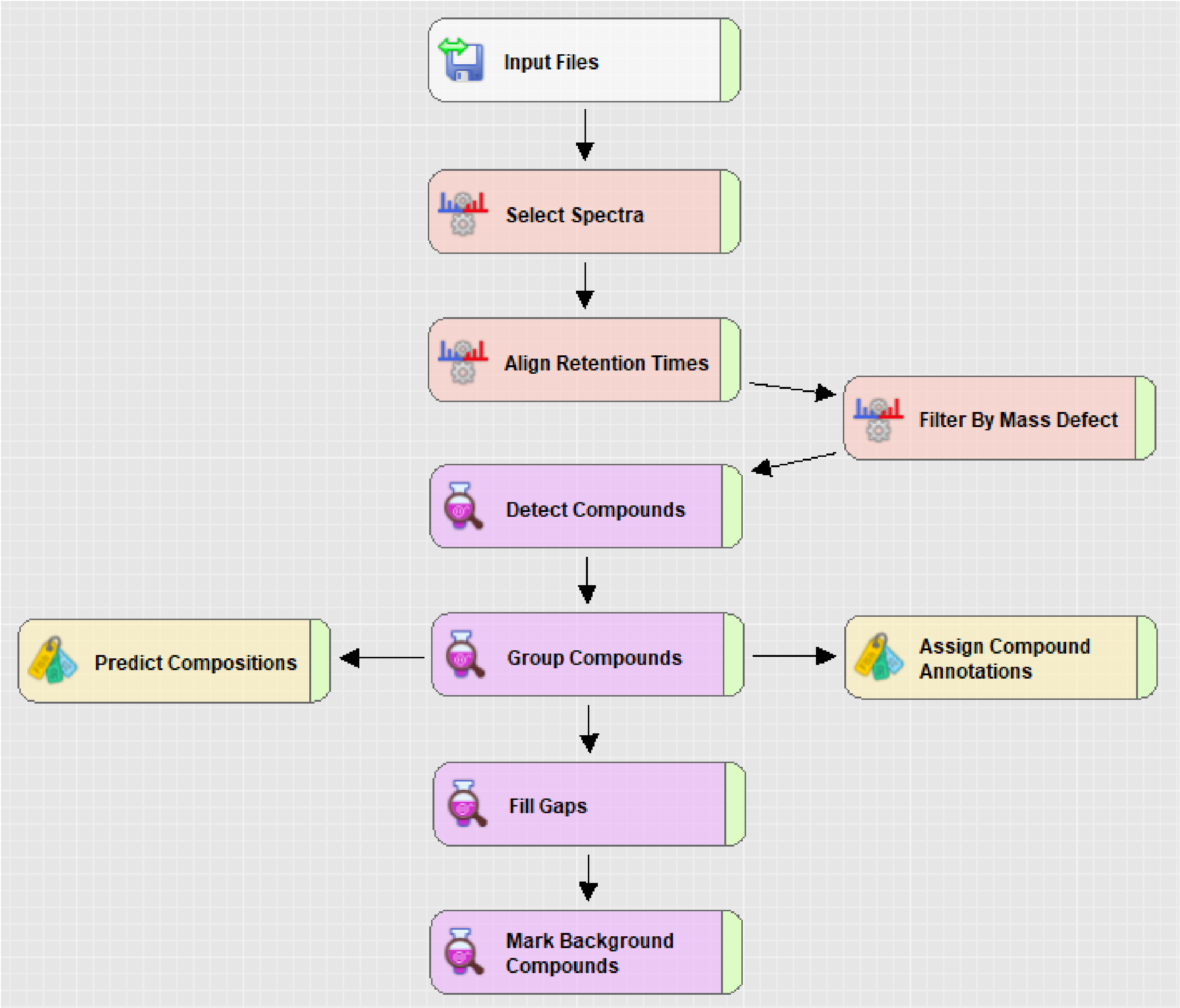

Figure S1. Scheme of the KMD filtering before "Compound Detection" tool (KMD before CD) workflow set up on Compound Discoverer software (v. 3.1) for minor phytocannabinoid analysis.

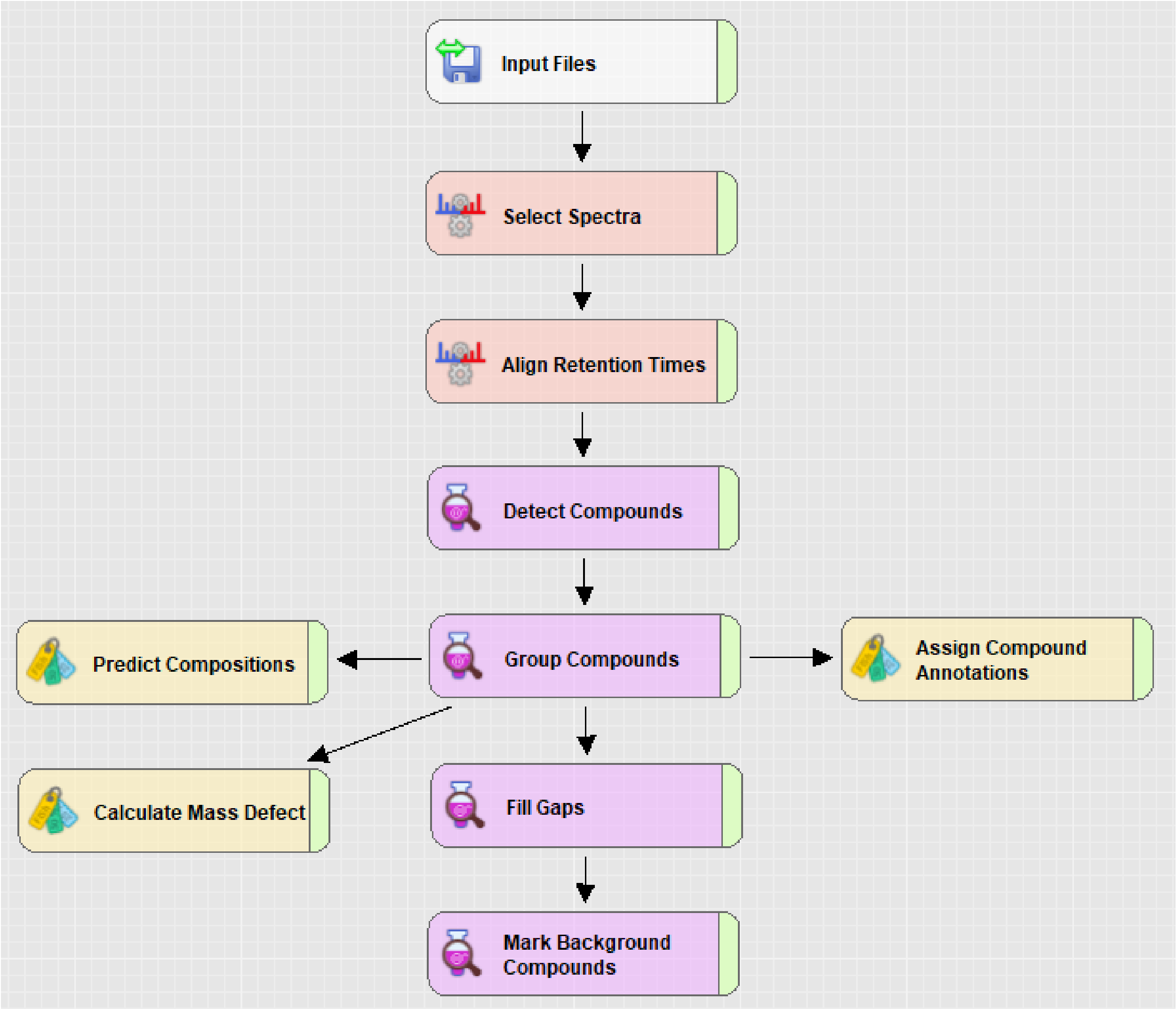

Figure S2. Scheme of the KMD filtering after "Compound Detection" tool (KMD after CD) workflow set up on Compound Discoverer software (v. 3.1) for minor phytocannabinoid analysis.

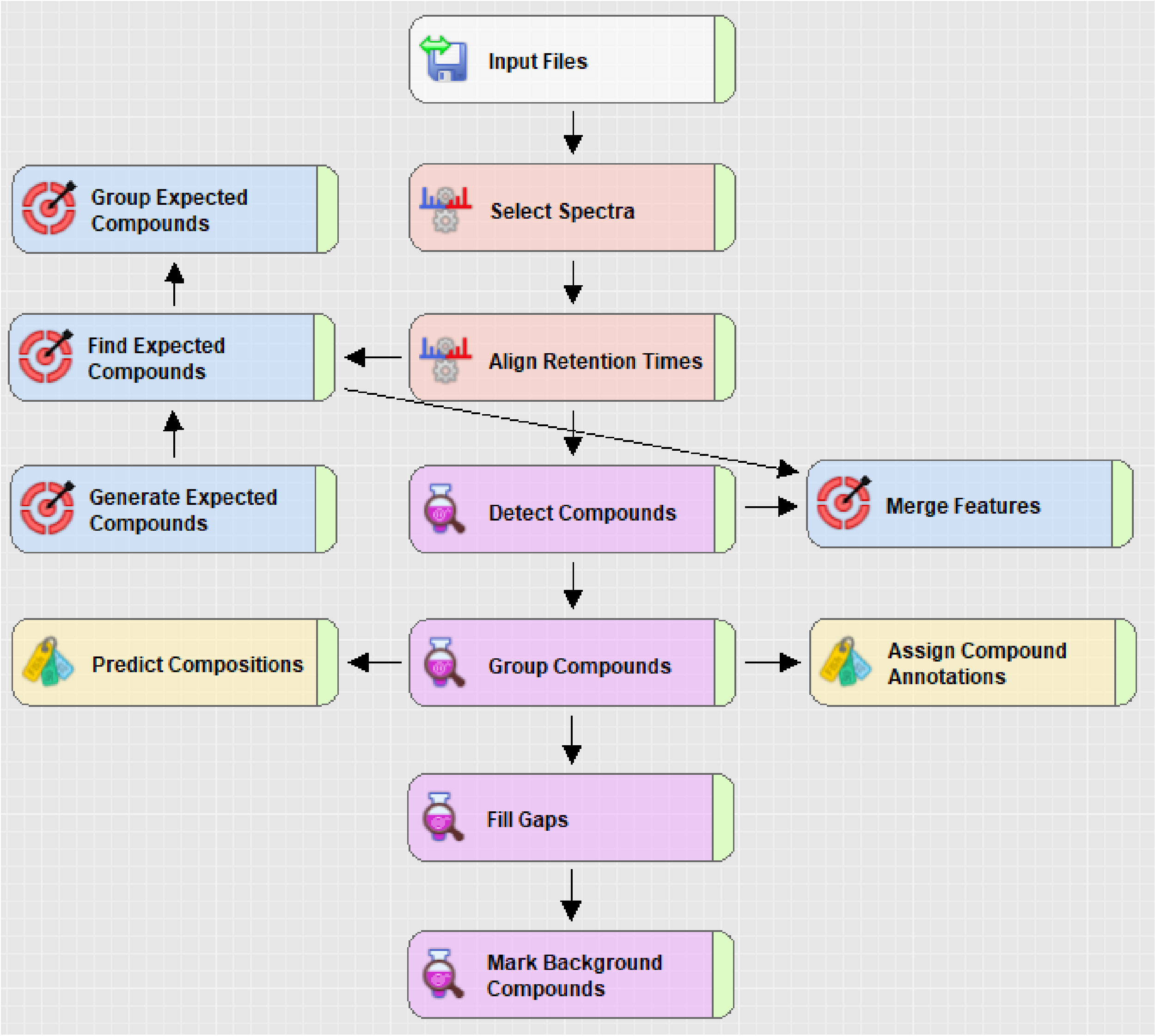

Figure S3. Scheme of the pseudo-KMD filtering using the "Expected Compounds" tool (pseudo-KMD with EC) workflow set up on Compound Discoverer software (v. 3.1) for minor phytocannabinoid analysis.

Table S2. List of the 61 annotated phytocannabinoids following the KMD before+after CD workflow and MS/MS spectral annotation, alongside their RT (min), proposed formula, molecular weights, Δmass (ppm), KMD, and diagnostic product ions.

Name	RT	Formula	Molecular Weight	Δmass	KMD	Diagnostic Product Ions
**CBNOA**	16.6	$C_{18}H_{18}O_4$	298.1208	0.9	-0.212	297.1133; 253.1233; 171.0807
**THCOA**	17.4	$C_{18}H_{22}O_4$	302.1517	-0.4	-0.186	301.1446; 283.1341; 257.1547; 189.0913; 135.0441; 123.0440
**CBDOA**	15.0	$C_{18}H_{22}O_4$	302.1519	0.4	-0.185	301.1446; 283.1341; 257.1547; 255.1390; 189.0913; 135.0441; 123.0440
**cis-THCOA**	17.1	$C_{18}H_{22}O_4$	302.1520	0.5	-0.185	301.1446; 283.1341; 257.1547; 189.0913; 135.0441; 123.0440
**CBCOA**	18.1	$C_{18}H_{22}O_4$	302.1520	0.7	-0.185	301.1446; 283.1341; 257.1547; 187.0757; 135.0441; 123.0440
**Δ^8-THCOA**	17.3	$C_{18}H_{22}O_4$	302.1521	0.9	-0.185	301.1446; 283.1341; 257.1547; 135.0441; 123.0440
**CBGOA**	15.7	$C_{18}H_{24}O_4$	304.1677	0.7	-0.172	303.1602; 285.1497; 259.1704; 189.0913; 135.0441; 123.0440
**CBD(C2)A**	15.5	$C_{19}H_{24}O_4$	316.1679	1.5	-0.185	315.1604; 297.1498; 271.1703; 269.1547; 203.1071; 149.0598; 137.0598
**CBC(C2)A**	18.7	$C_{19}H_{24}O_4$	316.1680	1.6	-0.185	315.1604; 297.1498; 271.1703; 201.0913; 149.0598; 137.0598
**THC(C2)A**	18.2	$C_{19}H_{24}O_4$	316.1680	1.6	-0.185	315.1604; 297.1498; 271.1703; 203.1071; 149.0598; 137.0598
**CBGOMA**	18.5	$C_{19}H_{26}O_4$	318.1835	1.1	-0.172	317.1753; 273.1861; 189.0913; 135.0441; 123.0440
**CBG(C2)A**	16.1	$C_{19}H_{26}O_4$	318.1838	2.0	-0.172	317.1757; 299.1654; 273.1862; 149.0598; 137.0598
**CBNVA**	18.2	$C_{20}H_{22}O_4$	326.1521	1.0	-0.212	325.1444; 281.1547; 171.0807
**CBNDVA**	14.2	$C_{20}H_{22}O_4$	326.1522	1.2	-0.212	325.1444; 281.1547; 171.0807
**CBLVA**	19.7	$C_{20}H_{26}O_4$	330.1833	0.7	-0.185	329.1758; 311.1653; 285.1860; 215.1074; 163.0755; 151.0754
**THCVA**	18.9	$C_{20}H_{26}O_4$	330.1834	0.7	-0.185	329.1758; 311.1653; 285.1860; 217.1229; 163.0755; 151.0754
**THCVA isomer**	14.6	$C_{20}H_{26}O_4$	330.1834	0.8	-0.185	329.1758; 311.1653; 285.1860; 217.1229; 163.0755; 151.0754
**cis-THCVA**	18.6	$C_{20}H_{26}O_4$	330.1834	0.8	-0.185	329.1758; 311.1653; 285.1860; 217.1229; 163.0755; 151.0754
**CBDVA isomer**	13.8	$C_{20}H_{26}O_4$	330.1834	0.9	-0.185	329.1758; 311.1653; 285.1860; 217.1229; 163.0755; 151.0754
**CBCVA**	19.4	$C_{20}H_{26}O_4$	330.1834	0.9	-0.185	329.1758; 311.1653; 285.1860; 215.1074; 163.0755; 151.0754

**THCVA isomer**	16.7	$C_{20}H_{26}O_4$	330.1834	0.9	-0.185	329.1758; 311.1653; 285.1860; 217.1229; 163.0755; 151.0754
**CBDVA**	16.1	$C_{20}H_{26}O_4$	330.1834	1.0	-0.185	329.1758; 311.1653; 285.1860; 283.1704; 217.1229; 163.0755; 151.0754
**CBGVA**	16.6	$C_{20}H_{28}O_4$	332.1991	1.0	-0.172	331.1916; 313.1812; 287.2018; 217.1229; 163.0755; 151.0754
**CBNBA**	19.1	$C_{21}H_{24}O_4$	340.1677	0.6	-0.212	339.1610; 295.1706
**CBDVMA**	18.8	$C_{21}H_{28}O_4$	344.1990	0.6	-0.185	343.1913; 299.2015; 231.1387; 165.0912; 150.0678
**THCBA**	19.8	$C_{21}H_{28}O_4$	344.1991	0.8	-0.185	343.1917; 325.1812; 299.2018; 231.1388; 177.0914; 165.0914
**cis-THCBA**	19.6	$C_{21}H_{28}O_4$	344.1991	0.9	-0.185	343.1917; 325.1812; 299.2018; 231.1388; 177.0914; 165.0914
**CBCBA**	20.2	$C_{21}H_{28}O_4$	344.1992	1.3	-0.185	343.1917; 325.1812; 299.2018; 229.1232; 177.0914; 165.0914
**CBDBA**	16.7	$C_{21}H_{28}O_4$	344.1992	1.3	-0.185	343.1917; 325.1812; 299.2018; 297.1861; 231.1388; 177.0914; 165.0914
**CBGVMA**	19.7	$C_{21}H_{30}O_4$	346.2147	0.8	-0.172	345.2072; 301.2174; 217.1229; 163.0755; 151.0754
**CBGBA**	17.1	$C_{21}H_{30}O_4$	346.2148	1.2	-0.172	345.2071; 327.1966; 301.2173; 231.1388;177.0914; 165.0914
**CBNA isomer**	18.7	$C_{22}H_{26}O_4$	354.1835	1.0	-0.212	353.1760; 309.1861
**CBNA**	19.9	$C_{22}H_{26}O_4$	354.1835	1.1	-0.212	353.1760; 309.1861; 171.0805
**CBNDA**	15.5	$C_{22}H_{26}O_4$	354.1837	1.7	-0.212	353.1760; 309.1861; 171.0805
**CBNDA isomer**	16.3	$C_{22}H_{26}O_4$	354.1839	2.2	-0.212	353.1760; 309.1861
**THCA isomer**	19.1	$C_{22}H_{30}O_4$	358.2147	0.7	-0.185	357.2074; 339.1967; 313.2175; 245.1547; 191.1070; 179.1069
**THCA isomer**	20.2	$C_{22}H_{30}O_4$	358.2147	0.7	-0.185	357.2074; 339.1967; 313.2175; 191.1070; 179.1069
**Δ^8-THCA**	20.5	$C_{22}H_{30}O_4$	358.2147	0.8	-0.185	357.2074; 339.1967; 313.2175; 245.1547; 191.1070; 179.1069
**THCA isomer**	19.7	$C_{22}H_{30}O_4$	358.2147	0.8	-0.185	357.2074; 339.1967; 313.2175; 245.1547; 191.1070; 179.1069
**THCA**	20.6	$C_{22}H_{30}O_4$	358.2147	0.9	-0.185	357.2074; 339.1967; 313.2175; 245.1547; 191.1070; 179.1069
**CBLA**	21.1	$C_{22}H_{30}O_4$	358.2147	0.9	-0.185	357.2074; 339.1967; 313.2175; 243.1386; 191.1070; 179.1069
**cis-THCA**	20.3	$C_{22}H_{30}O_4$	358.2148	1.0	-0.185	357.2074; 339.1967; 313.2175; 245.1547; 191.1070; 179.1069
**CBCA**	21.0	$C_{22}H_{30}O_4$	358.2148	1.0	-0.185	357.2074; 339.1967; 313.2175; 243.1386; 191.1070; 179.1069
**THCA isomer**	15.8	$C_{22}H_{30}O_4$	358.2148	1.0	-0.185	357.2074; 339.1967; 313.2175; 245.1547; 191.1070; 179.1069

**CBDA**	17.4	$C_{22}H_{30}O_4$	358.2148	1.0	-0.185	357.2074; 339.1967; 313.2175; 311.2019; 245.1547; 191.1070; 179.1069
**THCA isomer**	15.2	$C_{22}H_{30}O_4$	358.2148	1.1	-0.185	357.2074; 339.1967; 313.2175; 245.1547; 191.1070; 179.1069
**CBGA**	17.7	$C_{22}H_{32}O_4$	360.2304	0.9	-0.172	359.2230; 341.2123; 315.2330; 245.1547; 191.1070; 179.1069
**CBCHA**	21.7	$C_{23}H_{32}O_4$	372.2304	0.8	-0.185	371.2228; 353.2114; 327.2327; 205.1225; 193.1228
**THCHA**	21.4	$C_{23}H_{32}O_4$	372.2304	0.9	-0.185	371.2228; 353.2114; 327.2327; 259.1704; 205.1225; 193.1228
**CBDMA**	20.1	$C_{23}H_{32}O_4$	372.2304	0.9	-0.185	371.2229; 327.2330; 259.1704; 193.1227; 178.0991
**cis-THCHA**	21.2	$C_{23}H_{32}O_4$	372.2304	0.9	-0.185	371.2228; 353.2114; 327.2327; 205.1225; 193.1228
**CBDHA**	18.1	$C_{23}H_{32}O_4$	372.2306	1.5	-0.185	371.2228; 353.2114; 327.2327; 325.2175; 259.1704; 205.1225; 193.1228
**CBGMA**	20.9	$C_{23}H_{34}O_4$	374.2459	0.6	-0.172	373.2383; 329.2486; 245.1543; 191.1068; 179.1069
**CBGHA**	18.3	$C_{23}H_{34}O_4$	374.2461	1.1	-0.172	373.2389; 355.2283; 329.2489; 205.1225; 193.1228
**THCPA**	22.2	$C_{24}H_{34}O_4$	386.2462	1.2	-0.185	385.2387; 367.2280; 341.2485; 219.1386; 207.1384
**CBCPA**	22.5	$C_{24}H_{34}O_4$	386.2462	1.3	-0.185	385.2387; 367.2280; 341.2485; 219.1386; 207.1384
**CBDPA**	18.8	$C_{24}H_{34}O_4$	386.2463	1.5	-0.185	385.2387; 367.2280; 341.2485; 339.2330; 273.1862; 219.1386; 207.1384
**CBGHMA**	21.5	$C_{24}H_{36}O_4$	388.2618	1.2	-0.172	387.2546; 343.2640; 259.1703; 205.1225; 193.1228
**CBGPA**	18.9	$C_{24}H_{36}O_4$	388.2619	1.4	-0.172	387.2546; 369.2436; 343.2643; 219.1386; 207.1384
**SesquiCBGVA**	19.3	$C_{25}H_{36}O_4$	400.2620	1.7	-0.185	399.2549; 381.2435; 355.2640; 163.0755
**SesquiCBGA**	20.2	$C_{27}H_{40}O_4$	428.2931	1.0	-0.185	427.2856; 409.2751; 383.2959; 191.1072

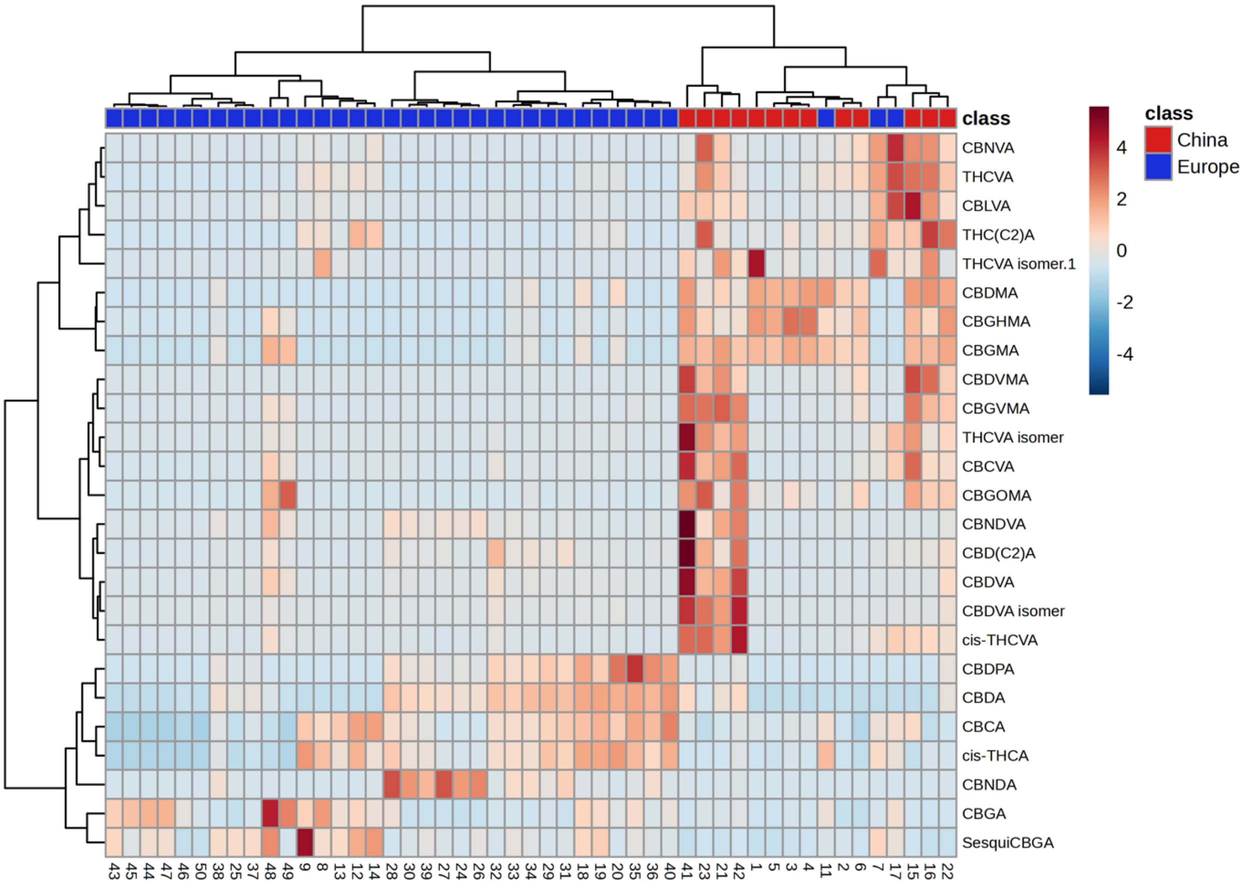

Figure S4. Hierarchical clustering heatmap and dendrogram built using the phytocannabinoid datasets grouping the samples based on the geographical origin of the seeds (Europe vs China).

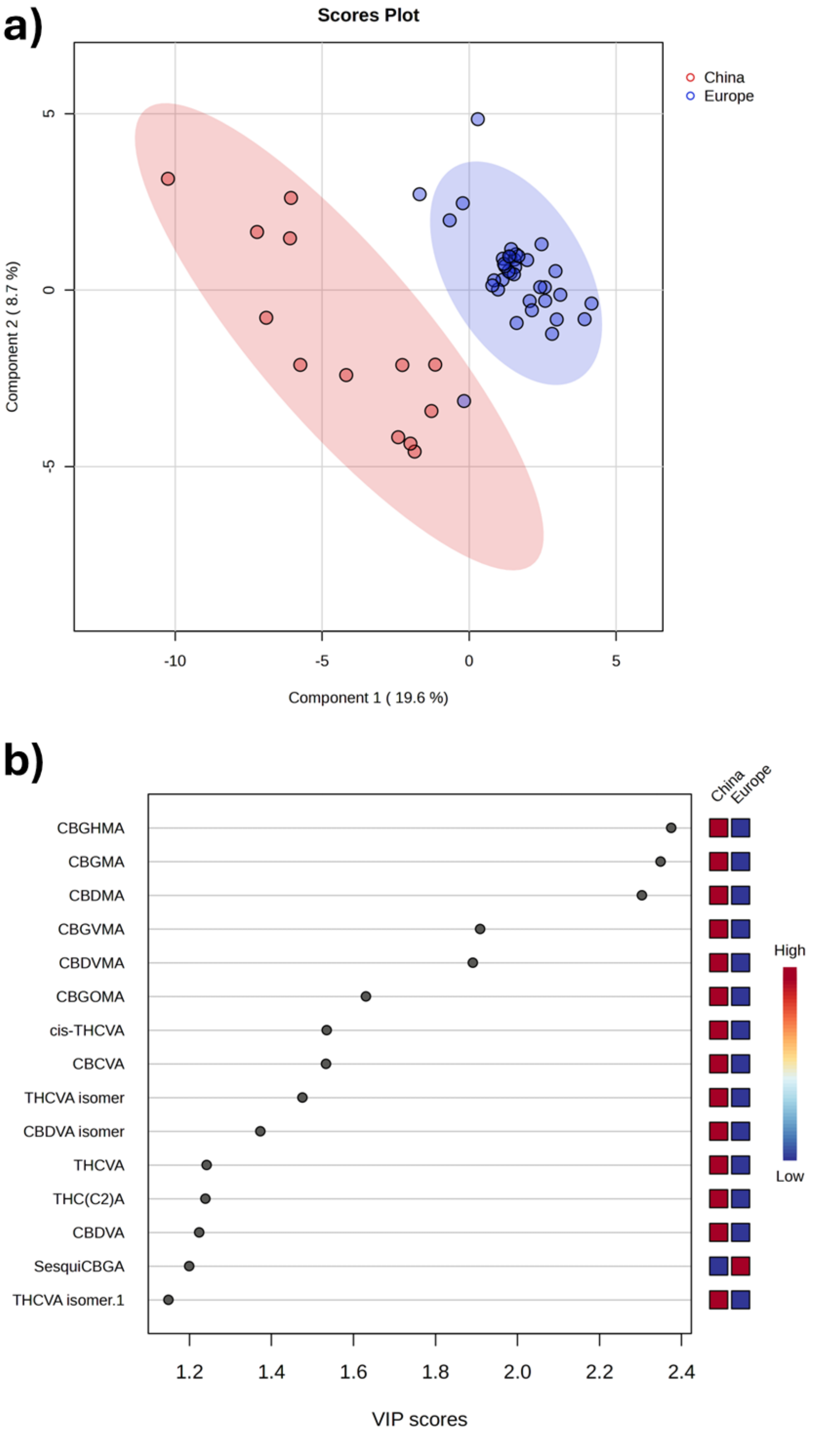

Figure S5. Partial least square-discriminant analysis (PLS-DA) built using the phytocannabinoid datasets grouping the samples based on the geographical origin of the seeds (Europe vs China) (a). Variable Importance in Projection (VIP) analysis based on the PLS-DA model (b).

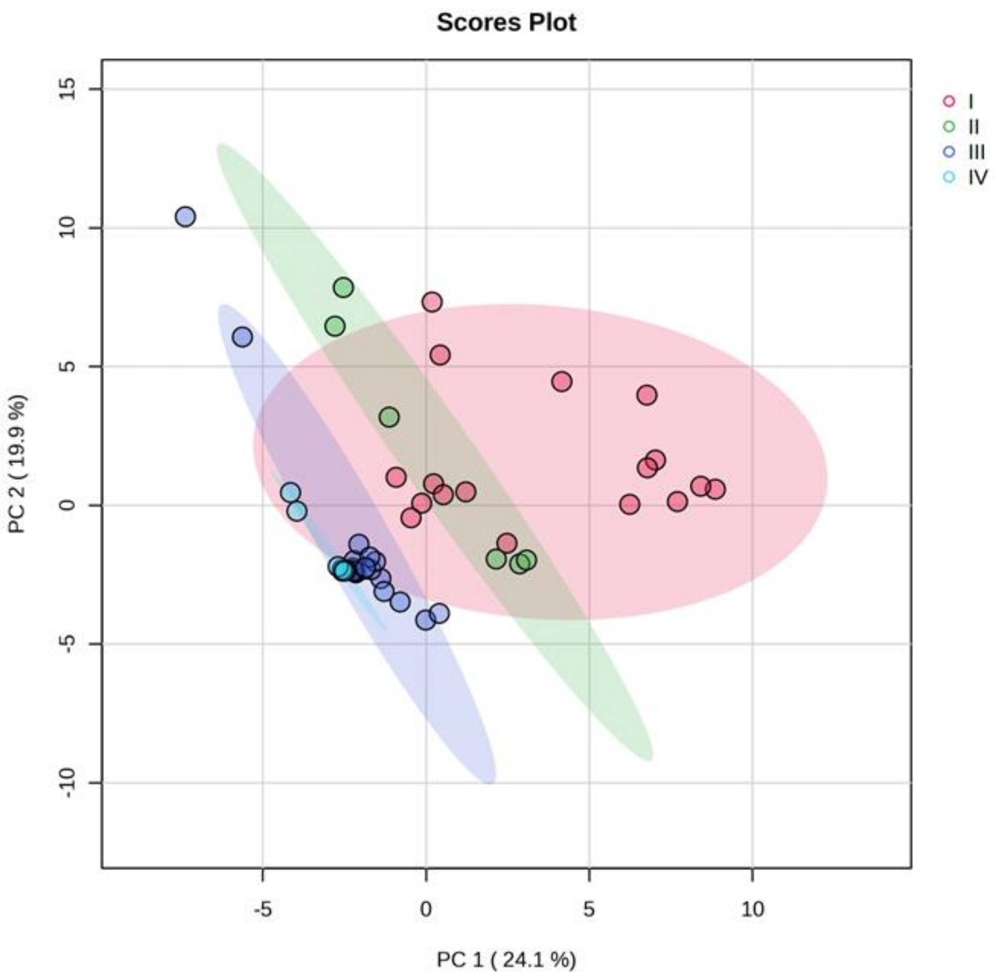

Figure S6. Principal Component Analysis (PCA) built using the phytocannabinoid datasets grouping the samples based on the chemovar of the samples.

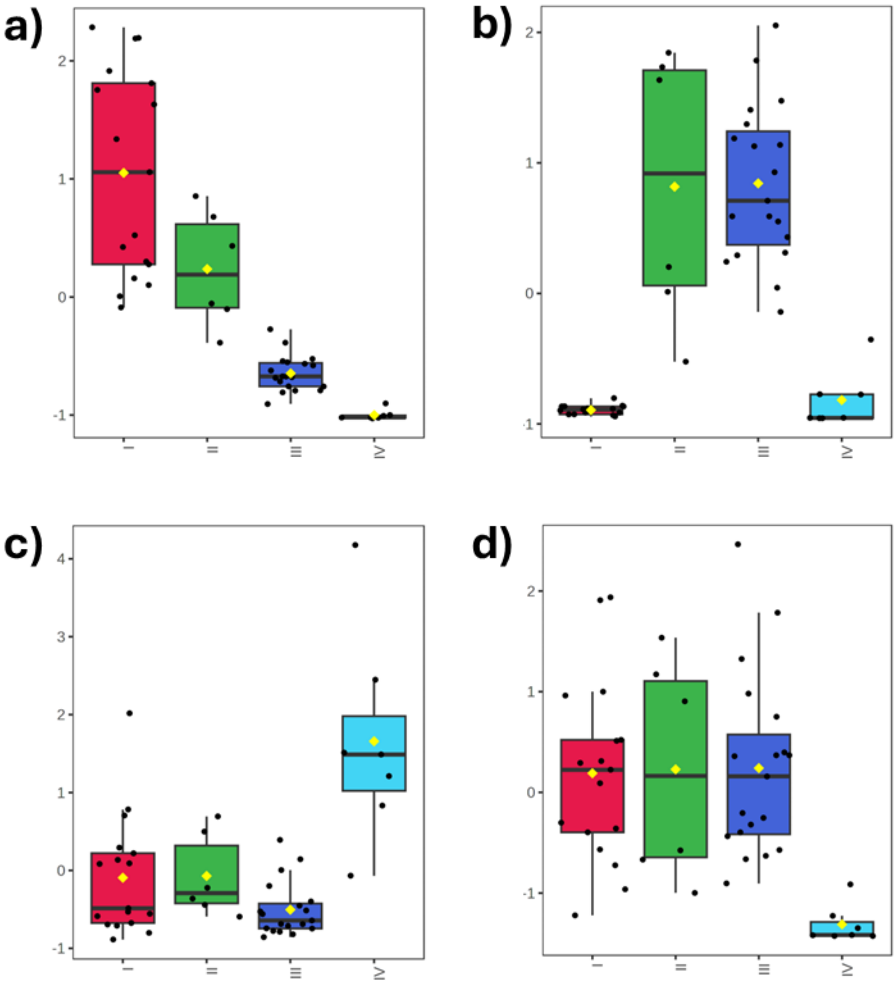

Figure S7. Box-and-whiskers plots of the major phytocannabinoids in the analyzed samples grouped in the four chemovars: (a) THCA, (b) CBDA, (c) CBGA, and (d) CBCA.

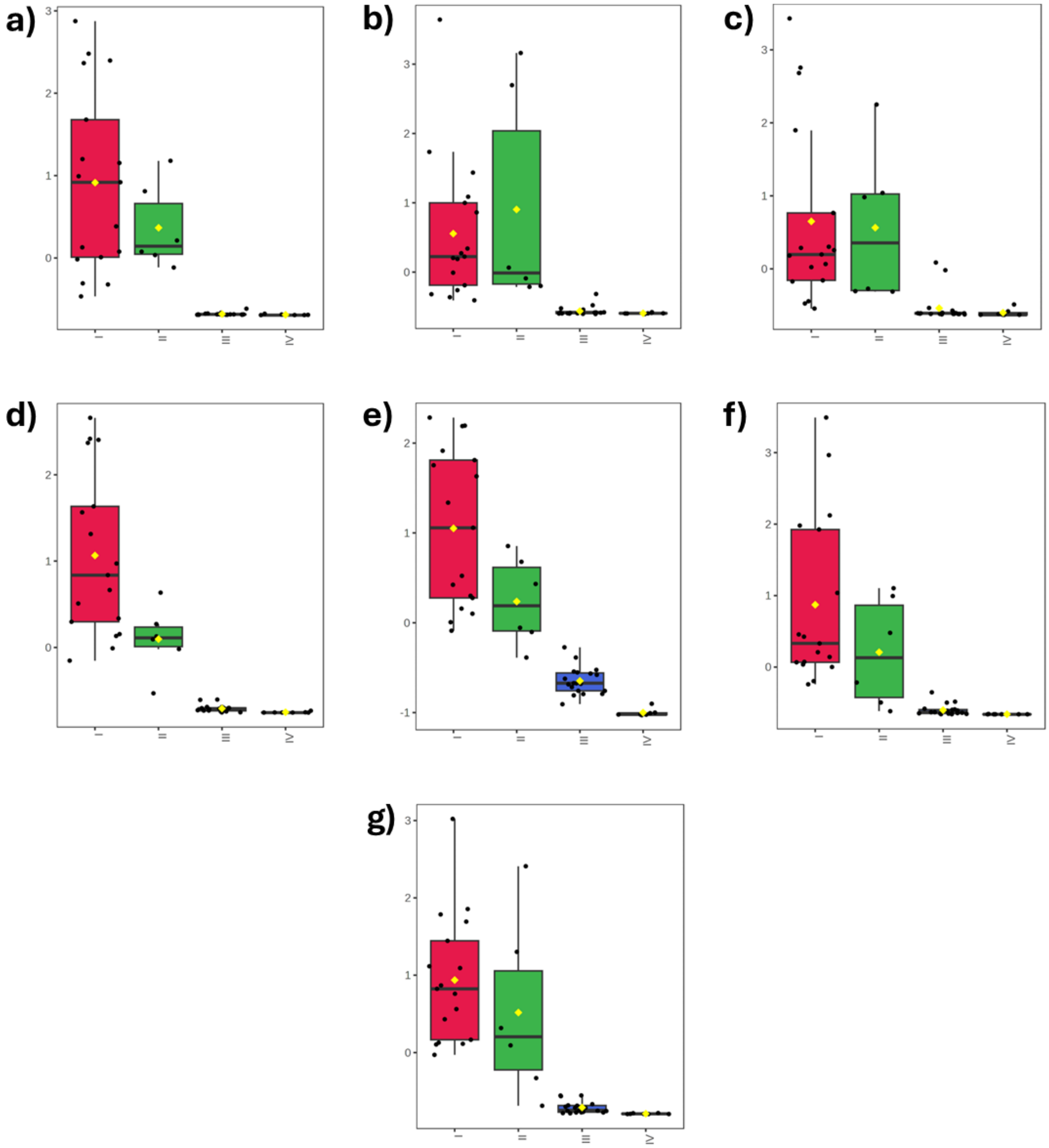

Figure S8. Box-and-whiskers plot of the THCA-type phytocannabinoids in the analyzed samples grouped in the four chemovars: (a) THCOA, (b) THC(C2)A, (c) THCVA, (d) THCBA, (e) THCA, (f) THCHA, and (g) THCPA.

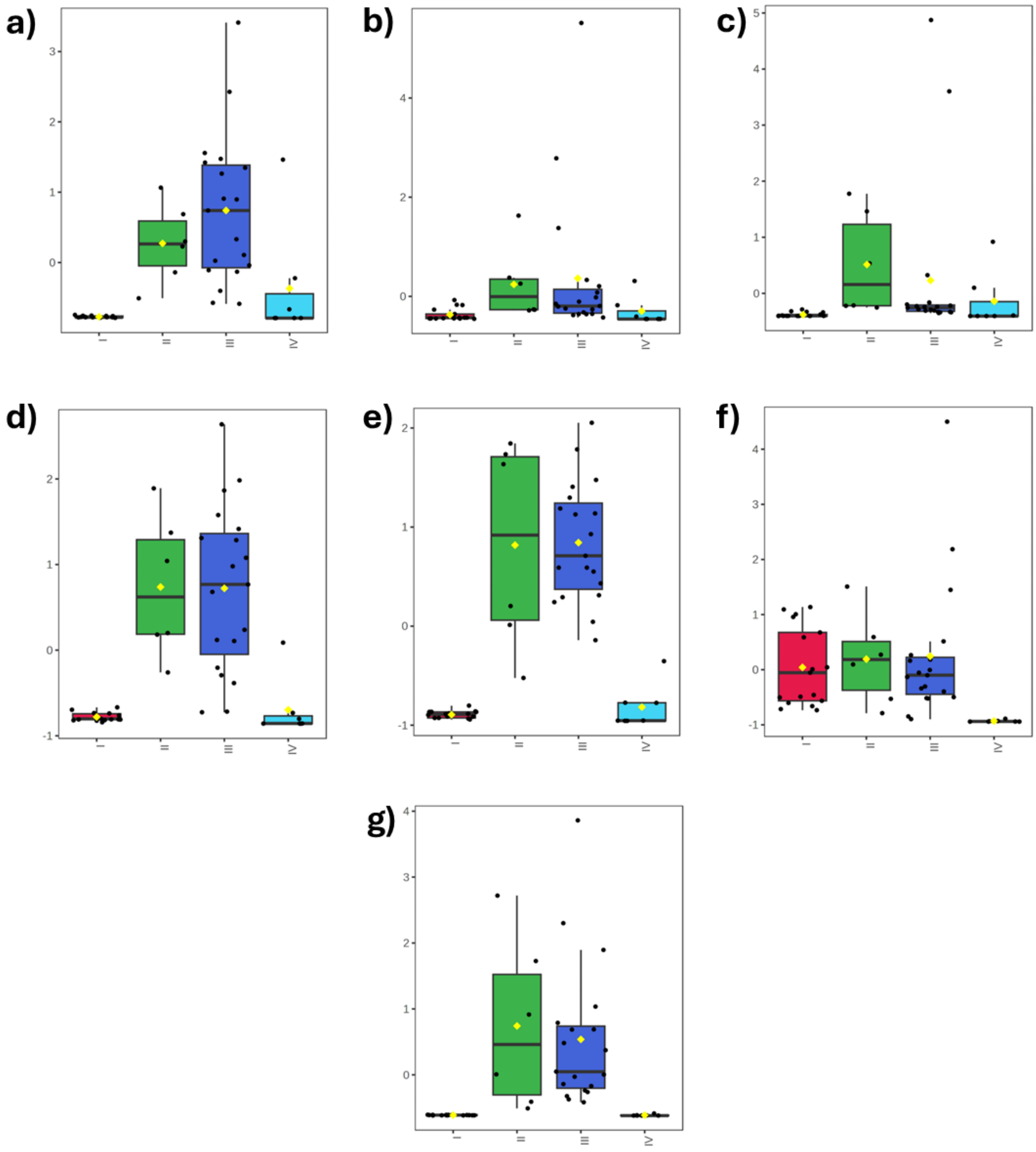

Figure S9. Box-and-whiskers plot of the CBDA-type phytocannabinoids in the analyzed samples grouped in the four chemovars: (a) CBDOA, (b) CBD(C2)A, (c) CBDVA, (d) CBDBA, (e) CBDA, (f) CBDHA, and (g) CBDPA.

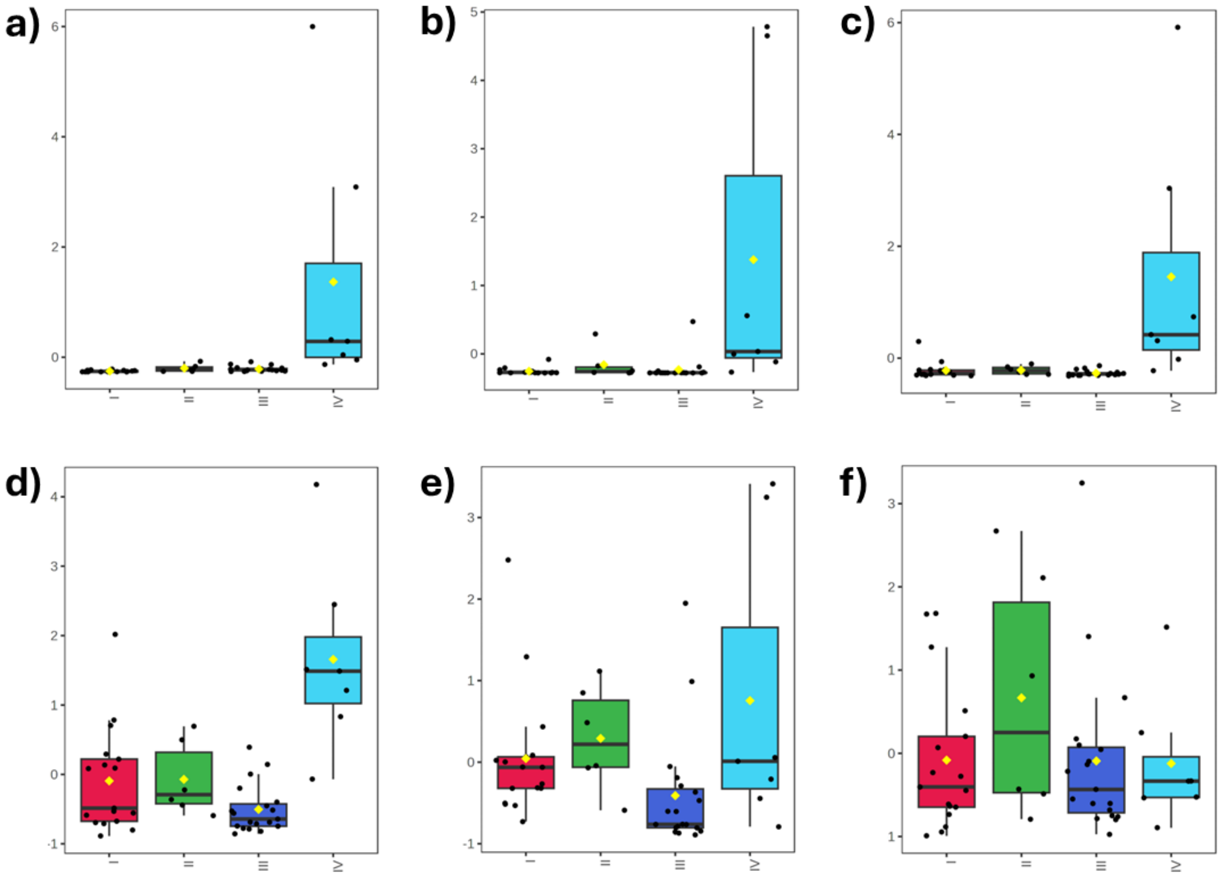

Figure S10. Box-and-whiskers plot of the CBGA-type phytocannabinoids in the analyzed samples grouped in the four chemovars: (a) CBDOA, (b) CBGVA, (c) CBGBA, (d) CBDA, (e) CBGHA, and (f) CBGPA.

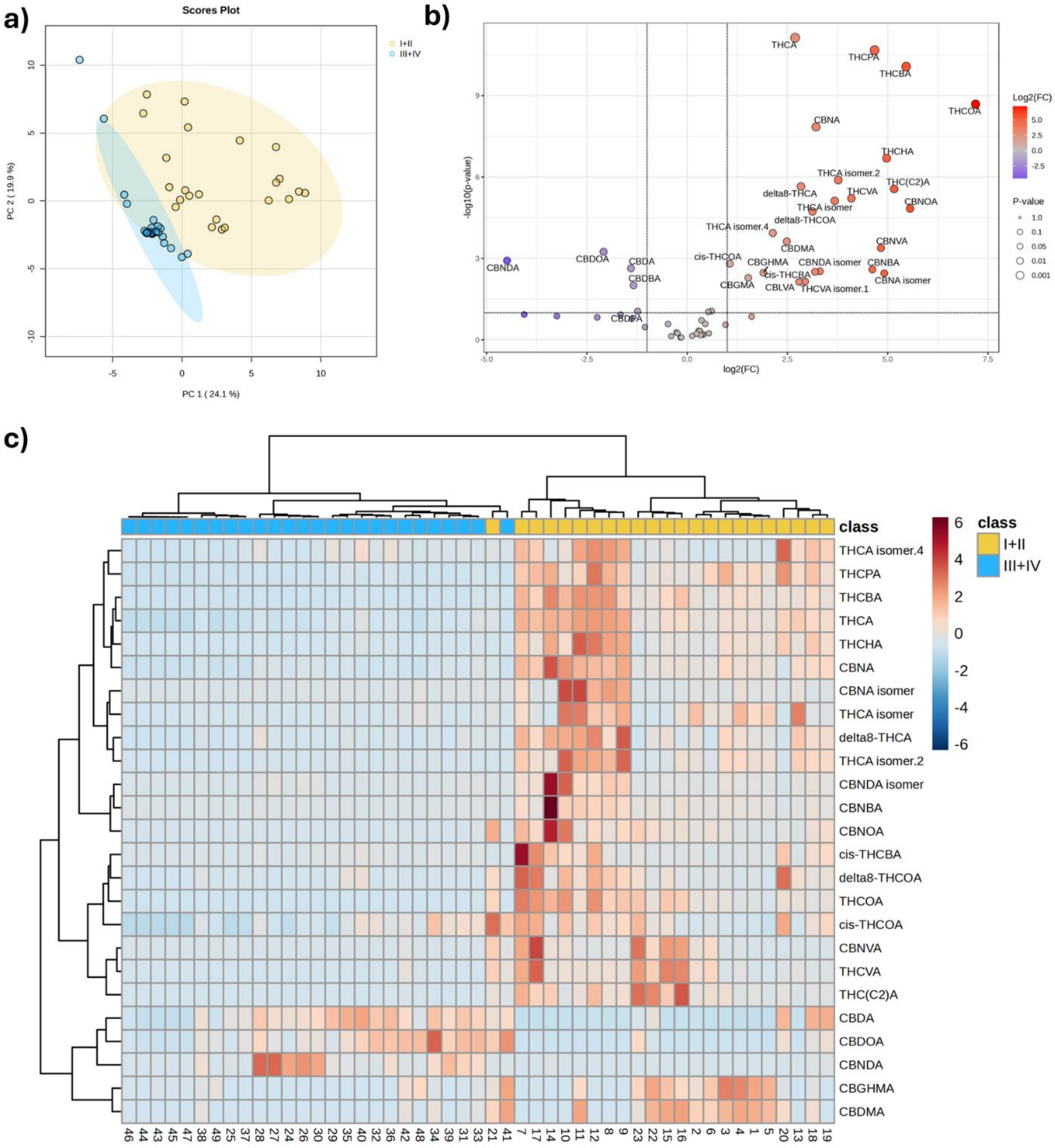

Figure S11. Principal Component Analysis (PCA, a), Volcano Plot Analysis (b), hierarchical clustering heatmap and dendrogram (c) built using the phytocannabinoid datasets grouping the samples in chemovar I+II and III+IV.

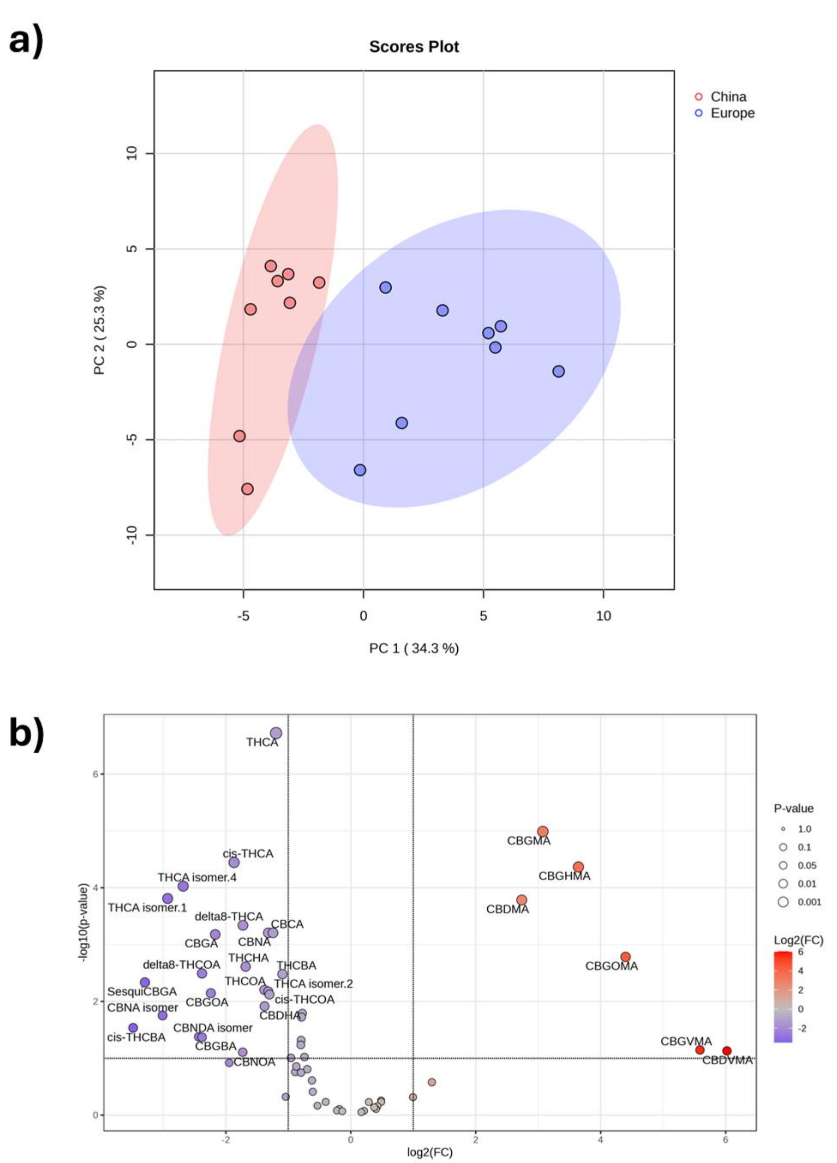

Figure S12. Principal component analysis (PCA, a) and Volcano plot analysis (b) built using the phytocannabinoid datasets of the samples from chemovar I based on their geographical origin (Europe vs China).

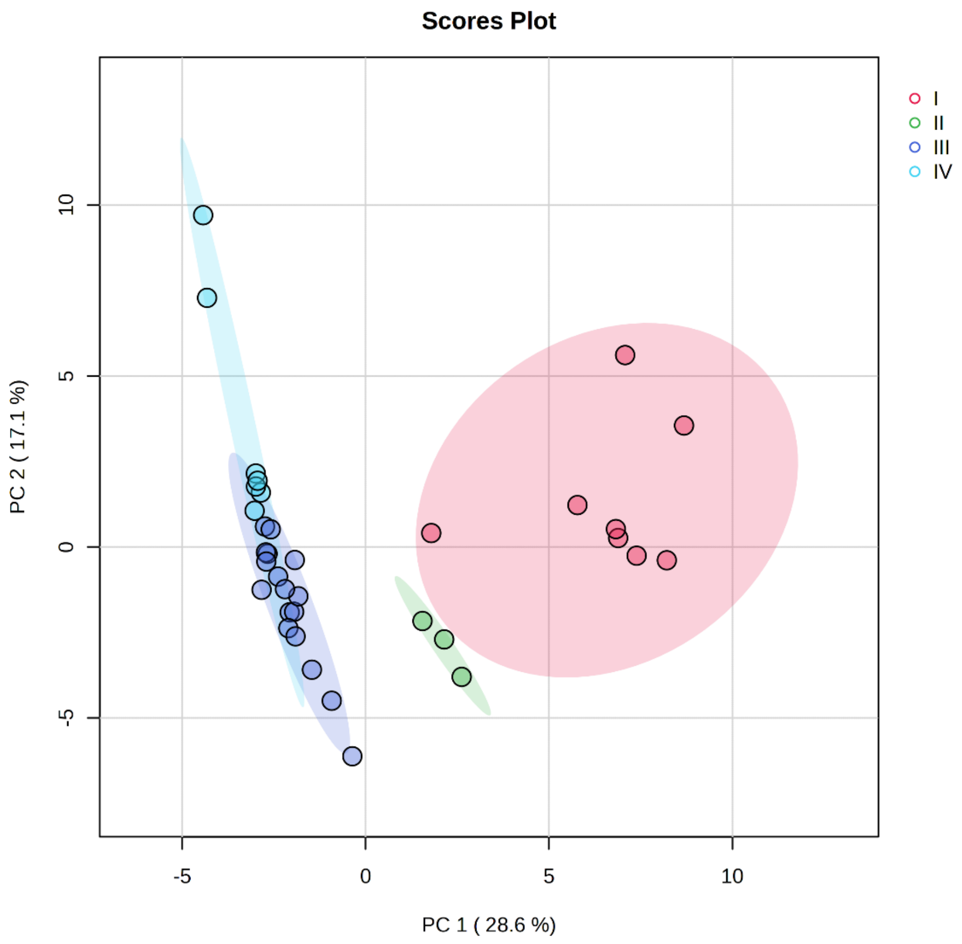

Figure S13. Principal Component Analysis (PCA) built using the phytocannabinoid datasets grouping the samples based on the chemovar of the samples whose seeds had European origin.

Table S3. THCA/THCVA and C5/C3 ratios based on the peak areas of the annotated phytocannabinoids from samples of chemovar I.

Sample	Origin	Chemotype	THCA/THCVA	C5/C3
**UN1**	China	I	8.5	12.9
**UN2**	China	I	1.4	2.3
**UN3**	China	I	8.0	14.4
**UN4**	China	I	3.1	5.3
**UN5**	China	I	13.4	21.6
**UN6**	China	I	0.8	1.1
**UN7**	Europe	I	1.2	1.8
**UN8**	Europe	I	4.1	11.7
**UN9**	Europe	I	4.7	10.7
**UN10**	America	I	3.2	6.1
**UN11**	Europe	I	4.4	7.9
**UN12**	Europe	I	4.5	9.4
**UN13**	Europe	I	5.1	11.9
**UN14**	Europe	I	4.8	9.2
**UN15**	China	I	0.5	0.8
**UN16**	China	I	0.5	0.8
**UN17**	Europe	I	0.6	1.3

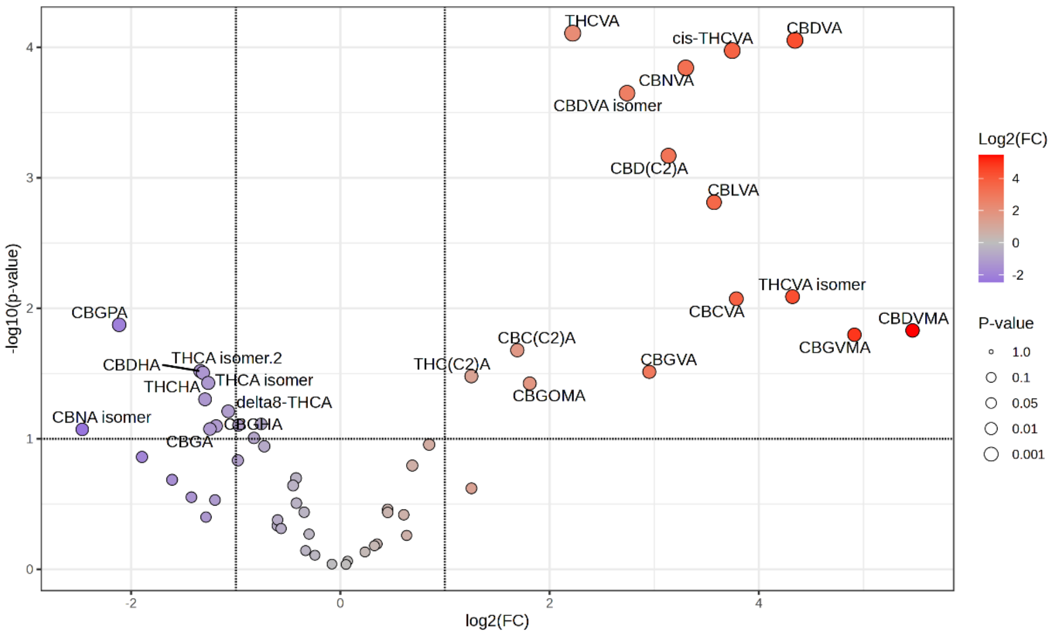

Figure S14. Volcano plot built using the phytocannabinoid datasets in the two defined subgroups of samples of chemovar I.

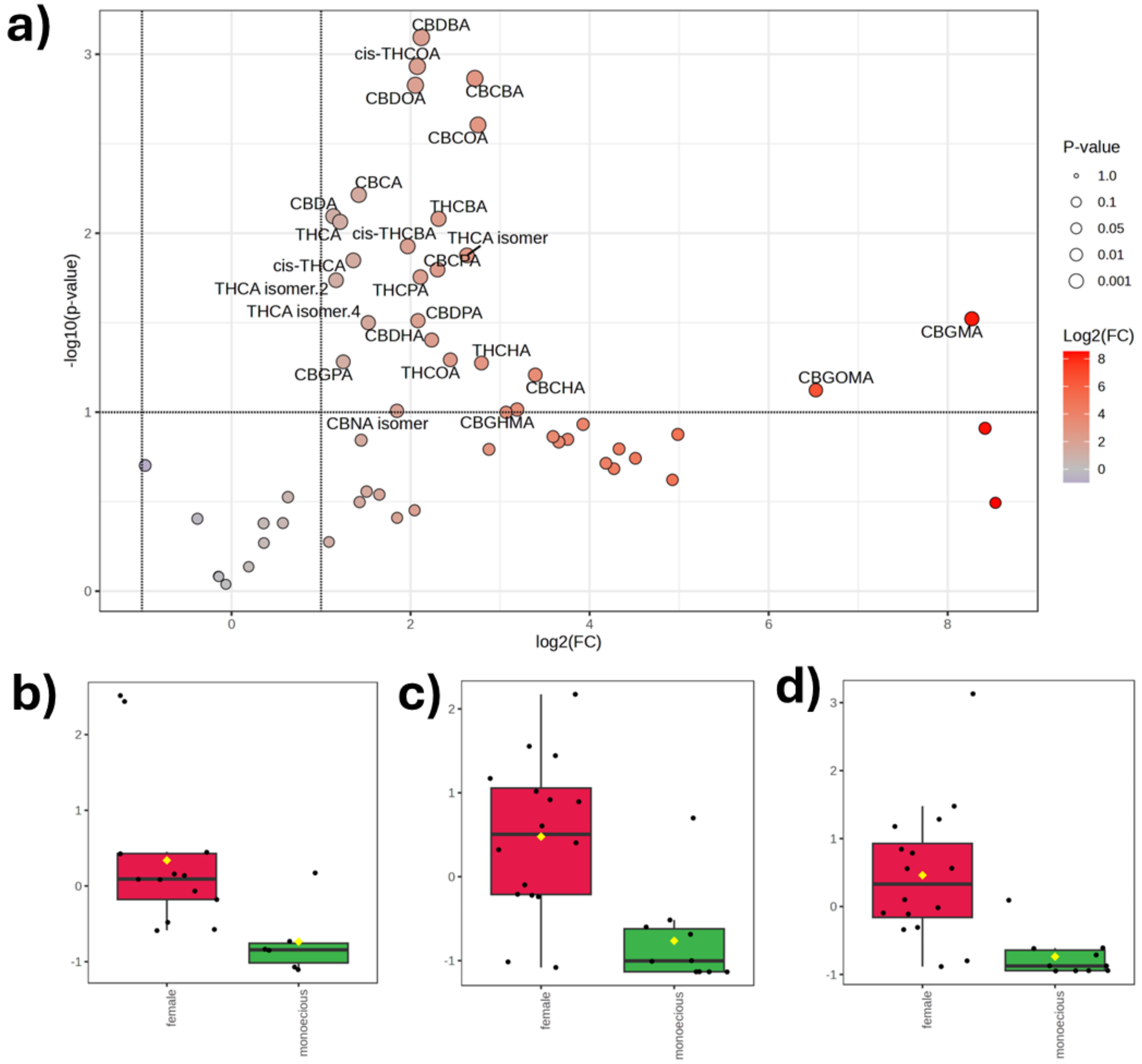

Figure S15. Volcano plot built using the phytocannabinoid datasets of samples of chemovars III and IV grouped based on the plant reproductive strategy (dioecious vs monoecious, a). Box-and-whiskers plots of (b) THCBA, (c) CBDBA, and (d) CBCBA in the two classes of cannabis samples based on the plant reproductive strategy.
